# Redox-Switchable Behavior of Transition-Metal Complexes Supported by Amino-Decorated N-Heterocyclic Carbenes

**DOI:** 10.3390/molecules27123776

**Published:** 2022-06-11

**Authors:** Mirko Ruamps, Stéphanie Bastin, Lionel Rechignat, Alix Sournia-Saquet, Laure Vendier, Noël Lugan, Jean-Marie Mouesca, Dmitry A. Valyaev, Vincent Maurel, Vincent César

**Affiliations:** 1 LCC-CNRS, Université de Toulouse, CNRS, 31077 Toulouse, France; mirko.ruamps@gmail.com (M.R.); stephanie.bastin@lcc-toulouse.fr (S.B.); lionel.rechignat@lcc-toulouse.fr (L.R.); alix.saquet@lcc-toulouse.fr (A.S.-S.); laure.vendier@lcc-toulouse.fr (L.V.); noel.lugan@laposte.net (N.L.); 2 Université Grenoble Alpes, CEA, CNRS, IRIG, SyMMES, 38000 Grenoble, France; jean-marie.mouesca@cea.fr

**Keywords:** redox-active ligands, N-heterocyclic carbene, organometallic complexes, EPR spectroscopy, DFT calculations

## Abstract

The coordination chemistry of the N-heterocyclic carbene ligand IMes^(NMe2)2^, derived from the well-known IMes ligand by substitution of the carbenic heterocycle with two dimethylamino groups, was investigated with d^6^ [Mn(I), Fe(II)], d^8^ [Rh(I)], and d^10^ [Cu(I)] transition-metal centers. The redox behavior of the resulting organometallic complexes was studied through a combined experimental/theoretical study, involving electrochemistry, EPR spectroscopy, and DFT calculations. While the complexes [CuCl(IMes^(NMe2)2^)], [RhCl(COD)(IMes^(NMe2)2^)], and [FeCp(CO)_2_ (IMes^(NMe2)2^)](BF_4_) exhibit two oxidation waves, the first oxidation wave is fully reversible but only for the first complex the second oxidation wave is reversible. The mono-oxidation event for these complexes occurs on the NHC ligand, with a spin density mainly located on the diaminoethylene NHC-backbone, and has a dramatic effect on the donating properties of the NHC ligand. Conversely, as the Mn(I) center in the complex [MnCp(CO)_2_ ((IMes^(NMe2)2^)] is easily oxidizable, the latter complex is first oxidized on the metal center to form the corresponding cationic Mn(II) complex, and the NHC ligand is oxidized in a second reversible oxidation wave.

## 1. Introduction

Since the isolation of the first free N-heterocyclic carbene (NHC) by Arduengo in 1991 [1], and the pioneering work of Herrmann in homogeneous catalysis as early as 1995 [2], NHCs were established as privileged ligands in organometallic chemistry and catalysis [3,4,5,6,7], and NHC-complexes found various promising applications as photoluminescent species [8] and metallodrugs [9], or in material chemistry [10]. This remarkable development was mainly driven by the aptitude of NHC ligands to stabilize transition metals in a wide range of oxidation states, due to their strong electronic donation properties, and to the good steric protection they offer [11,12]. Interestingly, while NHCs were first considered as fully innocent, pure-spectator ligands, the seminal report by Tumanskii and Apeloig in 2007 shows that imidazole-derived NHC ligands are also able to accept and delocalize radicals, or some spin density, within their heterocyclic scaffold [13], and, hence, that they could also be considered as redox-active ligands [14,15,16,17,18]. In parallel, redox-active NHC ligands were designed on purpose, by combining the carbenic heterocycle with some known redox-active moieties, such as ferrocene [19,20,21,22], cobaltocenium [23], quinone [24,25,26], *o*-aminophenols [27,28,29,30,31], or more recently, a naphthalene diimide backbone [32,33]. The redox-active NHC ligands allowed the design of redox-switchable catalysts by a strong modification of the electronic properties through a redox stimulus [7,34], or permitted the access to multiple oxidation states of the complexes.

**Figure 1:**
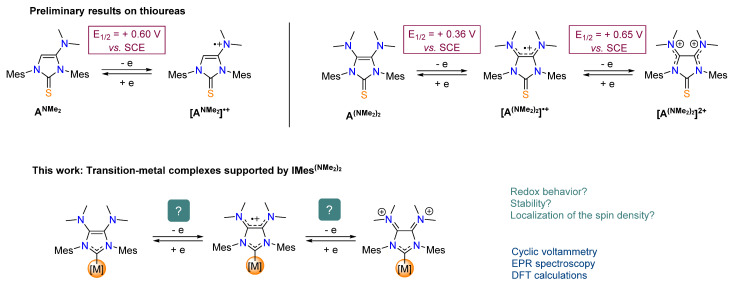
Redox behaviors of the thiourea **A^NMe^^2^** and **A^(NMe^^2)^^2^** derived from the amino-decorated IMes^NMe^^2^ and IMes^(NMe^^2)^^2^, respectively, and of the transition-metal complexes of IMes^(NMe^^2)^^2^, subject of the present study.

We entered this area of chemistry in 2018, by reporting preliminary studies on the redox behavior of the thiourea **A^NMe^^2^** and **A^(NMe^^2)^^2^**, derived from the mono-substituted 4-(dimethylamino)imidazol-2-ylidene IMes^NMe^^2^ and the di-substituted 4,5-bis(dimethylamino)imidazol-2-ylidene IMes^(NMe^^2)^^2^, respectively (Figure 1) [35]. While we studied the latter amino-derived NHCs mainly in the direction of organometallic catalysis with remarkable results [36,37,38,39,40], we actually initiated this program by considering the analogy between the NHC backbones and the electron-rich tri- and tetra-aminoethylene moieties, which are well-known electron-rich reducing agents [41,42]. We indeed show that both thioureas are redox-active, due to the presence of the dimethylamino groups onto their backbone. While **A^NMe^^2^** can be oxidized mono-electronically to the corresponding persistent radical-cation [**A^NMe^^2^**]^•**+**^, **A^(NMe^^2)^^2^** exhibits two oxidized forms, namely, the radical cation [**A^(NMe^^2)^^2^**]^•**+**^ and the diamagnetic dication [**A^(NMe^^2)^^2^**]**^2+^** and both are remarkably stable. Thus, we decided to extend this study to the case of the transition-metal complexes of the diamino-substituted IMes^(NMe^^2)^^2^ ligand (**1**), and we report herein that this NHC ligand exhibits redox-active behavior with a variety of metal centers.

## 2. Results and Discussion

### 2.1. Redox Properties of IMes^(NMe^^2)^^2^ Copper(I) Complex ***2***

**Figure 2:**
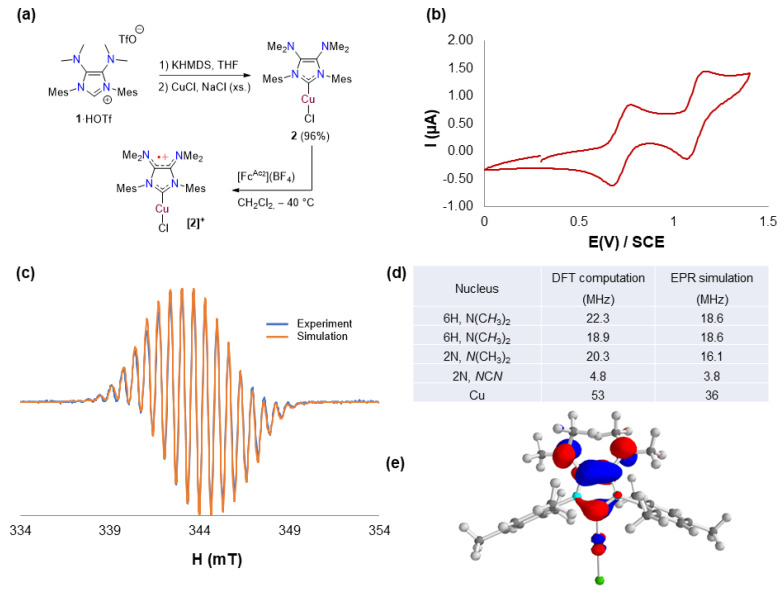
(**a**) Synthesis of complex **2** and subsequent chemical oxidation reaction to form **[2]^+^**. (**b**) Cyclic voltammogram of complex **2** (Pt working electrode, 1 mM in CH_2_Cl_2_ with 0.1 M Bu_4_NPF_6_ as the supporting electrolyte, 200 mV · s^−1^ scan rate; potentials are calibrated against Fc/Fc^+^ as internal standard with E_1/2_ (Fc/Fc^+^) = +0.46 V vs. SCE). (**c**) X-band EPR spectrum of **[2]^+^** in CH_2_Cl_2_/toluene solution (1:1) at room temperature: microwave frequency = 9.6508 GHz, mod. ampl. = 0.1 mT, power = 2 mW. Simulation parameters: g_iso_ = 2.00338, lw = 0.05 + 0.1 mT (Gaussian + Lorentzian) (**d**) Isotropic hyperfine coupling constants obtained by DFT computation and by simulation. (**e**) DFT-optimized structure of **[2]^+^**, and representation of the SOMO (isodensity value of 0.05 a.u.).

We started our investigation by studying the behavior of the copper(I) complex [CuCl(**1**)] (**2**), which is synthesized by reacting the free stable NHC **1**, generated from **1**·HOTf by deprotonation with one equivalent of potassium bis(trimethylsilyl)amide (KHMDS), with an excess of CuCl and NaCl at room temperature (Figure 2a). These unusual conditions are necessary to avoid the competitive formation of the cationic homoleptic complex [Cu(**1**)_2_]^+^ under standard conditions (1 equivalent of CuCl) [43]. The ^1^H NMR spectrum of **2** is particularly simple, as only four singlets are observed, due to the *C*_2v_ symmetry of the molecule, and to the equivalence of the twelve protons of the NMe_2_ substituents, as already observed in the previous complexes of ligand **1** [36,39,40]. The redox behavior of complex **2** was first examined by cyclic voltammetry (CV) measurements (Figure 2b), and compared to the parent unsubstituted complex [CuCl(IMes)] (Figure S1). While complex [CuCl(IMes)] displays no clear oxidation wave, the bis-amino-substituted complex **2** features two fully reversible one-electron transfer processes at E_1/2_ = +0.71 V, and +1.11 V vs. SCE. This CV pattern is highly similar to that observed for the corresponding thiourea **A^(NMe^^2)^^2^** [35], with the exception that both oxidation processes in **2** occur at higher potentials. This suggests that the redox events in complex **2** might also be located on the NHC ligand, and prompted us to explore in more detail the mono- and di-oxidized complexes [**2**]**^+^** and [**2**]**^2+^**.

Complex **2** was chemically oxidized using an equimolar amount of 1,1′-diacetylferrocenium tetrafluoroborate ([Fc^(Ac)^^2^](BF_4_), E_1/2_ = +0.95 V [44]) in CH_2_Cl_2_ at −40 °C, to give a deep blue solution of the one-electron oxidized species **[2]^+^**, which was characterized immediately by EPR spectroscopy at room temperature in CH_2_Cl_2_/toluene (1:1) solution (Figure 2c). The X-band EPR spectrum of **[2]^+^** is consistent with a delocalized organic radical (Landé factor of g_iso_ = 2.00338), and displays a symmetrical, hyperfine splitting pattern. The simulated spectrum fits perfectly with the experimental spectrum, and is composed mainly of hyperfine interactions between the radical and twelve equivalent protons (a_H_ = 18.6 MHz), with the two exocyclic nitrogen atoms (a_N_ = 16.1 MHz) and with the copper center (a_Cu_ = 36 MHz), and, to a lower extent, by a hyperfine coupling constant with the two nitrogen atoms of the diaminocarbene unit (a_N_ = 3.8 MHz) (Figure 2d). The optimized geometry computed by DFT for **[2]**^•**+**^ confirms that the copper center remains linear and dicoordinate, which is in agreement with a Cu(I) center, and shows, in particular, a planarization of the two exocyclic nitrogen atoms (Σ_N_ = 359.5° and 359.6°), with a twist of the NMe_2_ plane relative to the carbenic heterocycle of ca. 34°, most probably due to steric congestion, as already encountered in the related thiourea derivative [**A^(NMe^^2)^^2^**]^•**+**^. Similarly, the SOMO orbital of **[2]^+^** is mainly localized on the bis(dimethylamino)ethylene backbone, involving C−N π-antibonding and C−C π-bonding characters, with small contributions of the carbene and copper centers (Figure 2e). The DFT-computed hyperfine coupling (hfc) constants are consistent with the hfc values obtained from the simulation of the EPR spectrum. The DFT calculation distinguishes two groups in particular for the hydrogen atoms from the NMe_2_ groups, depending on whether they are localized on the inner or outer methyl groups, and gives two distinct hfc coupling constants of a_H_ = 22.3 MHz and a_H_ = 18.9 MHz. Eventually, the mono-oxidized complex **[2]^+^** is shown to be stable in solution for 2–3 h at room temperature. 

We then tried to generate the di-oxidized complex **[2]^2+^**. Due to the high potential observed for the second oxidation wave in CV (E_1/2_ = +1.11 V vs. SCE), strong oxidizing agents were employed. Thus, complex **2** was reacted at a low temperature (−50 °C) with 2.5 equivalents of [N(*p*-BrPh)_3_](SbF_6_) (Magic Blue, E_1/2_ = +1.16 V vs. SCE) or [NO](SbF_6_) (E_1/2_ = +1.46 V vs. SCE), but only mixtures of unidentified products were detected by NMR, and even if the reaction mixture was indeed diamagnetic, no definite conclusion could be drawn on the nature of **[2]^2+^**.

Overall, this first study confirms that NHC ligand **1** behaves as a redox-active ligand in organometallic complexes, and that the mono-oxidized complex **[2]^+^** is generated and characterized by EPR spectroscopy.

### 2.2. Redox Properties of IMes^(NMe^^2)^^2^ Dicarbonyl Rhodium(I) Complex ***3***

We then turned our attention to the rhodium carbonyl complex [RhCl(CO)_2_(**1**)] (**3**) that we had previously synthetized [36], with the aim of evaluating the overall donor properties of the NHC by means of IR spectroscopy [11]. 

**Figure 3:**
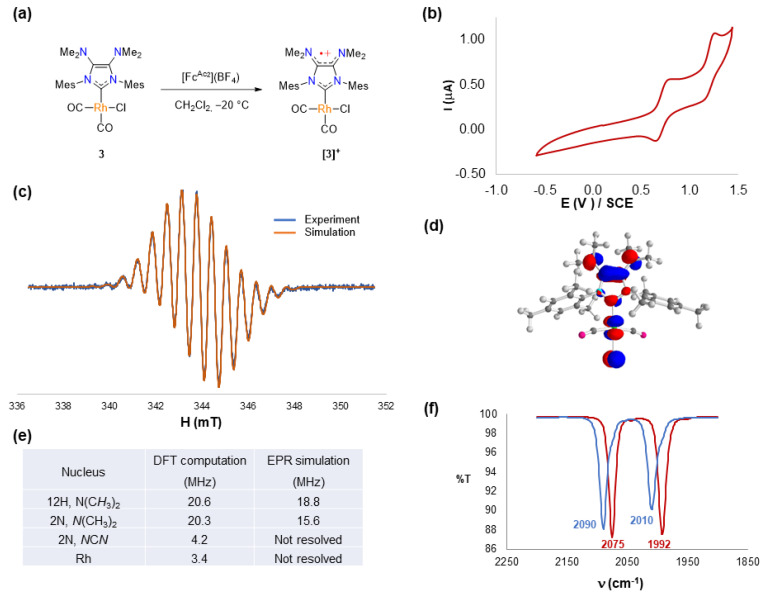
(**a**) Chemical oxidation of complex **3** into **[3]^+^**. (**b**) Cyclic voltammogram of complex **3** (Pt working electrode, 1 mM in CH_2_Cl_2_ with 0.1 M Bu_4_NPF_6_ as the supporting electrolyte, 200 mV · s^−1^ scan rate; potentials are calibrated against Fc/Fc^+^ as internal standard with E_1/2_ (Fc/Fc^+^) = +0.46 V vs. SCE). (**c**) X-band EPR spectrum of **[3]^+^** in CH_2_Cl_2_/toluene solution (1:1) at room temperature: microwave frequency = 9.65225 GHz, mod. ampl. = 0.05 mT, power = 5 mW. Simulation parameters: g_iso_ = 2.00354, lw = 0.4 mT (Gaussian). (**d**) DFT-optimized structure of **[3]^+^**, and representation of the SOMO (isodensity value of 0.05 a.u.). (**e**) Isotropic hyperfine coupling constants obtained by DFT computation and by simulation. (**f**) IR spectra of complexes **3** (in red) and **[3]^+^** (in blue) in 2250–1850 cm^−1^ range in CH_2_Cl_2_ solution.

The cyclic voltammogram of complex **3** displays a first reversible oxidation wave at E_1/2_ = +0.71 V vs. SCE, and a second irreversible oxidation process at E_pa_ = +1.26 V vs. SCE (Figure 3b), whose reversibility may be restored at higher scan rate (10 V · s^−1^). Interestingly, the first oxidation wave of **3** occurs at the same potential as for complex **2**, and, hence, corresponds to an oxidation of the NHC backbone. To confirm the nature of the mono-oxidized state **[3]^+^**, we then carried out the chemical oxidation of **3** using one equivalent of 1,1′-diacetylferrocenium tetrafluoroborate in CH_2_Cl_2_ at −20 °C (Figure 3a), and analyzed **[3]^+^** by EPR spectroscopy in CH_2_Cl_2_/toluene solution (1:1) at room temperature. Unlike the Cu(I) compound **[2]^+^**, the Rh(I) complex **[3]^+^** is stable under these conditions only for several minutes. Analogously to **[2]^+^**, the EPR spectrum of **[3]^+^** features a symmetrical hyperfine coupling pattern (Figure 3c), which is perfectly simulated, taking into account hyperfine interactions between the single electron and the twelve hydrogen atoms of the NMe_2_ groups (a_H_ = 18.8 MHz) and with the two exocyclic nitrogen atoms (a_N_ = 15.6 MHz). These values are very close to those calculated for **[2]^+^**, and are corroborated by DFT calculations, which give similar hfc values for those interactions. In addition, the DFT calculations indicate two much smaller hyperfine couplings with the two endocyclic nitrogen atoms and with the ^103^Rh center (I = ½), which were not resolved in the experimental spectrum. The experimental and simulated EPR spectra and the computed SOMO orbital in **[3]^+^** (Figure 3d) are, thus, in agreement with an organic radical delocalized on the bis(dimethylamino)ethylene backbone of the NHC ligand IMes^(NMe^^2)^^2^, leaving the Rh(I) center untouched, as already observed for the thiourea **A^(NMe^^2)^^2^** and the Cu(I) complex **2**. Therefore, it is possible to quantify the effect of the mono-oxidation of the IMes^(NMe^^2)^^2^ ligand on its electronic properties by recording the IR spectra of complexes **3** and **[3]^+^**, as the average frequency of the two ν_CO_ bands in the complexes [RhCl(CO)_2_(NHC)] in CH_2_Cl_2_ is linearly correlated to the Tolman electronic parameter (TEP) value of the NHC ligand [45,46]. While the carbonyl stretching in **3** are measured at 1992 and 2075 cm^−1^ (TEP = 2047 cm^−1^), the two bands shift to higher frequencies upon oxidation to **[3]^+^** and are observed at 2010 and 2090 cm^−1^ (TEP = 2060 cm^−1^). 

**Figure 4:**
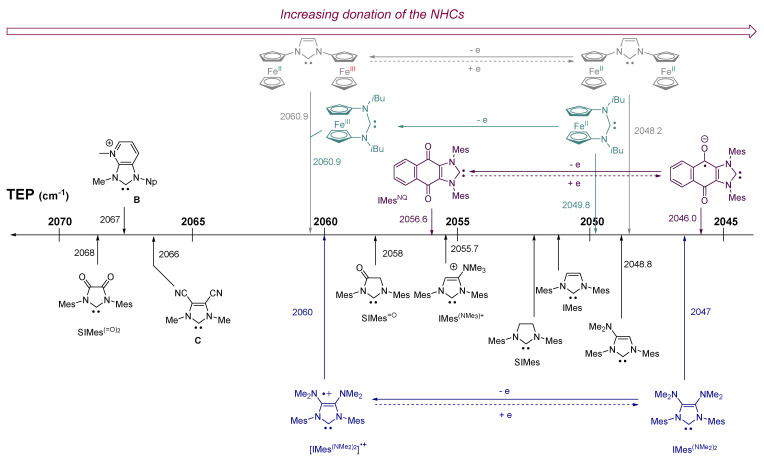
TEP value scale of selected, relevant NHCs.

A scale of TEP values of selected, relevant NHC ligands is depicted in Figure 4. While the ligand IMes^(NMe^^2)^^2^ is a better donor than the regular IMes, the mono-oxidized cationic radical [IMes^(NMe^^2)^^2^]^•+^ is a quite weak electron-donor, and is an even weaker donor than the naphthoquinone-annelated IMes^NQ^ (TEP = 2056.6 cm^−1^) [22,47], the (amino)(amido)carbene SIMes^=O^ (TEP = 2058 cm^−1^) [48], and the cationic IMes^(NMe^^3)+^ (TEP = 2055.7 cm^−1^) [49]. However, it remains a greater donor than the least electron-donating NHC ligands represented by the diamidocarbene SIMes^(=O)^^2^ (TEP = 2068 cm^−1^) [50], the cationic NHC **B** (TEP = 2067 cm^−1^) [51], or the dicyano-substituted NHC **C** (TEP = 2067 cm^−1^) [52]. Interestingly, the difference in TEP values of 13 cm^−1^ between the two redox states of IMes^(NMe^^2)^^2^ indicates that the latter constitutes a new class of redox-switchable NHC ligand, with the previous examples relying mainly on the incorporation of redox-active ferrocene-derived moiety, or of a naphthoquinone, into the NHC skeleton [7,53]. Moreover, the relatively high shift between the two oxidation states IMes^(NMe^^2)^^2^ / [IMes^(NMe^^2)^^2^]^•+^ can be explained by the fact that the redox event in IMes^(NMe^^2)^^2^ occurs directly onto the carbenic heterocycle and, thus, has a strong effect on the electron-donating properties of the carbenic center.

### 2.3. Redox Properties of Half-Sandwich IMes^(NMe^^2)^^2^ Fe(II) and Mn(I) Complexes ***[4]***(BF_4_) and ***5***

**Scheme 1:**
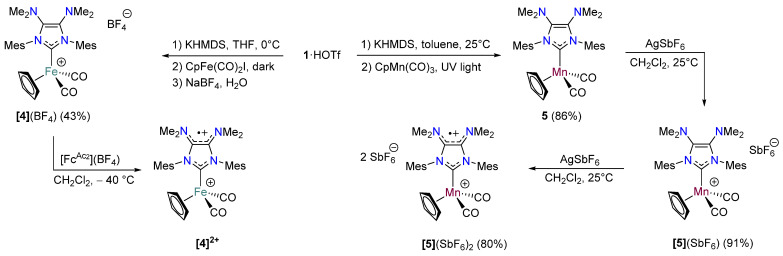
Synthesis of the Fe(II) complex **[4]**(BF_4_), and chemical generation of the oxidized Fe(II) complex **[4]^2+^** (**left**). Synthesis of Mn(I) complex **5**, and its stepwise oxidation to Mn(II) derivatives **[5]**(SbF_6_) and **[5]**(SbF_6_)_2_ (**right**).

**Figure 5:**
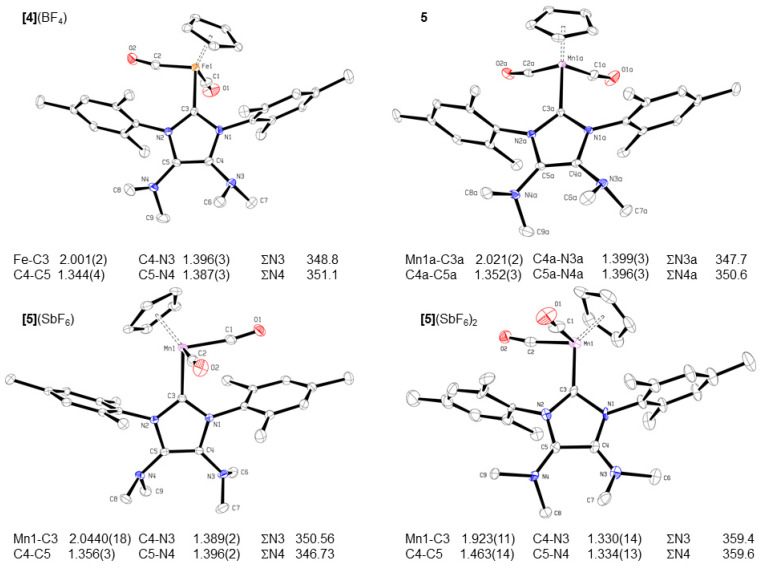
Molecular structures of half-sandwich complexes **[4]**(BF_4_), **5**, **[5]**(SbF_6_), and **[5]**(SbF_6_)_2_, and selected bond lengths (Å) and angles (deg). Ellipsoids drawn at 30% probability level. Hydrogen atoms and counter-anions omitted for clarity.

The study continued with the two isoelectronic d^6^, 18e-valence electron half-sandwich complexes [CpFe(CO)_2_(**1**)] ](BF_4_) (**[4]**(BF_4_), and [CpMn(CO)_2_(**1**)] (**5**) of Fe(II), and Mn(I), respectively. Complex **[4]**(BF_4_) was synthesized through a slight modification of the synthetic procedure reported by Guerchais for [CpFe(CO)_2_(IMes)]I [54]. The free stable carbene **1** was first reacted with the precursor [CpFe(CO)_2_I] in the dark to generate the intermediate [CpFe(CO)_2_(**1**)]I, and the iodide anion was then exchanged in situ for the non-coordinating tetrafluoroborate anion BF_4_^−^, to yield the complex **[4]**(BF_4_) in 43% isolated yield (Scheme 1). It is of note that the anion metathesis is found to be crucial for the photostability of complex **[4]**(BF_4_). The Mn(I) complex [CpMn(CO)_2_(**1**)] (**5**) was synthesized according to a classical UV light-induced substitution of one carbonyl by the free carbene **1** from cymantrene [CpMn(CO)_3_] [55], and was isolated in very good yield. Both complexes were fully characterized by spectroscopic and analytical methods, and their molecular structures firmly established by single-crystal X-ray diffraction analysis (Figure 5). Beyond the expected piano-stool coordination geometries around the metal and the metal−C_NHC_ bond lengths, which are within the classical range for such complexes [54,55], the C4−C5 bond lengths are characteristic of a double C=C bond (1.35 Å) [56], and the pyramidalization of the two exocyclic nitrogen atoms (Σ_N_ ~ 350°) indicates that the nitrogen lone pairs do not interact with the π-system of the carbenic heterocycle, as previously observed in other complexes [36,39,40].

**Figure 6:**
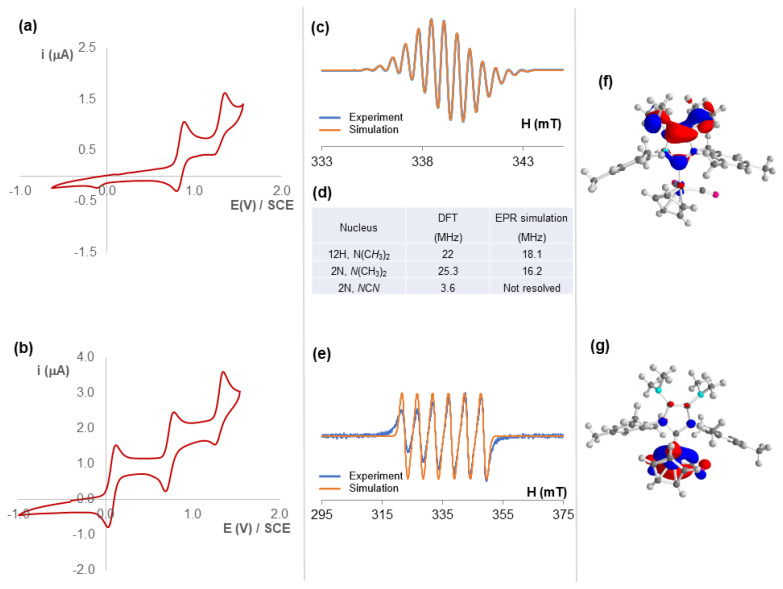
(**a**,**b**) Cyclic voltammograms of complexes **4** and **5**, respectively (Pt working electrode, 1 mM in CH_2_Cl_2_ with 0.1 M Bu_4_NPF_6_ as the supporting electrolyte, 200 mV · s^−1^ scan rate; potentials are calibrated against Fc/Fc^+^ as internal standard with E_1/2_ (Fc/Fc^+^) = +0.46 V vs. SCE). (**c**) X-band EPR spectrum of **[4]^2+^** in CH_2_Cl_2_/toluene solution (1:1) at room temperature: microwave frequency = 9.5042 GHz, mod. ampl. = 0.1 mT, power = 5 mW. Simulation parameters: g_iso_ = 2.0006, lw = 0.43 mT (Gaussian) (**d**) Isotropic hyperfine coupling constants obtained by DFT computation and by simulation for **[4]^2+^**. **(e)** X-band EPR spectrum of **[5]^+^** in CH_2_Cl_2_/toluene solution (1:1) at room temperature: microwave frequency = 9.6513 GHz, mod. ampl. = 0.5 mT, power = 2 mW. Simulation parameters: g_iso_ = 2.0537, a_Mn_ = 150.2 MHz, and lw = 1.98 mT (Gaussian). (**f**,**g**) DFT-optimized structures for **[4]^2+^** and **[5]^+^**, respectively, and representation of SOMO (isodensity value of 0.05 a.u.).

While complexes **[4]**(BF_4_) and **5** are isoelectronic, their cyclic voltammograms differ substantially (Figure 6). The iron complex **[4]**(BF_4_) shows a similar electrochemical behavior to those of previously studied derivatives **2** and **3,** with a reversible oxidation wave at E_1/2_ = +0.84 V vs. SCE, and an irreversible second oxidation process at E_pa_ = +1.36 V vs. SCE, which indicates that the NHC ligand **1** is still the location where the redox process takes place. Conversely, the much more electron-rich Mn(I) complex **5** exhibits three oxidation processes at E_1/2_ = +0.08 V, +0.75 V, and +1.33 V vs. SCE, with the two first being fully reversible and the latter only partially. The potential at +0.08 V vs. SCE of the first additional oxidation wave lies in the typical range of potentials for the oxidation of Mn(I) to Mn(II) centers in a series of [CpMn(CO)_2_(NHC)] reported by us [57,58] and others [59], and, thus, corresponds to a metal-centered oxidation process. The lower potential of this process for **5** (E_1/2_ = +0.08 V vs. SCE), compared to the process for the parent [CpMn(CO)_2_(IMes)] (E_1/2_ = +0.16 V vs. SCE), is also consistent with the more electron-donating character of ligand **1,** relative to the IMes ligand.

These first assumptions are confirmed by the study of the mono-oxidized species **[4]^2+^** and **[5]^+^**. The Fe-complex **[4]^2+^** is generated chemically by oxidation with 1,1′-diacetylferrocenium tetrafluoroborate, and the resulting red/purple solution analyzed by X-band EPR and IR spectroscopies at room temperature (Figure 6). The same hyperfine coupling pattern is observed as encountered for the previous complexes **[2]^+^** and **[3]^+^**, and is perfectly fitted by hyperfine couplings with the 12 hydrogen atoms (a_H_ = 18.1 MHz) and the two nitrogen atoms (a_N_ = 16.2 MHz) of the exocyclic NMe_2_ groups. IR monitoring of **[4]**(BF_4_) oxidation in CH_2_Cl_2_ shows the partial formation of new species **[4]^2+^**, evidenced by the appearance of ν_CO_ bands at 2054 and 2015 cm^−1^, which gradually decompose at room temperature, precluding its isolation. The average ν_CO_ value for **[4]^2+^** exhibits a ~12 cm^−1^ higher frequency shift relative to that of the starting product, thus, showing similar behavior as observed in case of Rh(I) complexes **3**/**[3]^+^** (*vide supra*). The calculated SOMO of **[4]^2+^** is also in agreement with the experimental data, and confirms that the radical is mainly localized on the bis(dimethylamino)ethene backbone of ligand **1**. The Mn(I) complex **5** is readily oxidized using one equivalent of AgSbF_6_ to yield the oxidized complex **[5]**(SbF_6_), which is isolated in pure form in 91% yield as black crystals. The X-band EPR spectrum of **[5]^+^** recorded at room temperature is consistent with a Mn(II) metal center, as the Landé factor of g_iso_ = 2.0537 deviates significantly from an organic radical, and the spectrum is composed of only six equidistant peaks arising from the hyperfine coupling between the unpaired electron and the ^55^Mn nucleus (I = 5/2, abundance = 100%, a_Mn_ = 150.2 MHz). This Mn(I)-to-Mn(II) transformation in **[5]**(SbF_6_) is also confirmed by IR (Figure S2), UV–vis (Figure S3), and paramagnetic ^1^H NMR spectroscopy, exhibiting similar features to the previously observed [Cp(CO)_2_Mn(IMes)](BF_4_) analogue [54], as well as by an X-ray diffraction analysis of single crystals (Figure 4). Finally, DFT calculations of **[5]^+^** show that the SOMO is mainly located on the Mn center. In particular, the molecular structure of **[5]**(SbF_6_) shows that the metrics within the NHC ligand **1** remain about the same compared to the non-oxidized complex **5**, especially on the diaminoethene backbone. As expected for such Mn(II) NHC complexes, the Mn1−C3 bond [2.0440(18) Å] (DFT *in vacuo*: 2.015 Å) is a little elongated, relative to the Mn1—C3a bond in **5** [2.021(2) Å] [57].

The full reversibility of the second oxidation wave in the CV of **5** then prompted us to explore the oxidation of **[5]**(SbF_6_). The latter is cleanly oxidized by using one more equivalent of AgSbF_6_ in CH_2_Cl_2_ to give a dark blue solution, from which the dicationic complex **[5]**(SbF_6_)_2_ is isolated as a black powder in 80% yield. In contrast to the first metal-centered oxidation of complex **5,** leading to ca. 120 cm^−1^ high frequency shift of ν_CO_ bands, the average stretching CO frequencies are much less affected upon the **[5]^+^** → **[5]^2+^** transformation (+7.5 cm^−1^), being consistent with the occurrence of electron transfer at the NHC ligand. The intense absorption at 578 nm observed in the UV–vis spectrum of **[5]**(SbF_6_)_2_ (Figure S3) is similar to that observed for the radical cation of [**A^(NMe^^2)^^2^**]^•**+**^ (590 nm), previously attributed to the electronic transition involving SOMO orbital by TD-DFT calculations [32]. Important changes are worth mentioning in the molecular structure of **[5]**(SbF_6_)_2_ as compared to **[5]**(SbF_6_) and **5** (Figure 5), especially on the NHC backbone. Indeed, the two exocyclic nitrogen atoms are no longer pyramidalized but planar [Σ_N3_ = 359.4°, Σ_N4_ = 359.6°], the C4−C5 bond of the imidazolyl ring is elongated [+0.11 Å] relative to C4−C5 bonds in **5** and **[5]^+^**, and the C4−N3 and C5−N4 bonds are shortened [*ca.* −0.06 Å] relative to the corresponding bonds in **5** and **[5]^+^**. This is consistent with a π-delocalization through the bis(dimethylamino)ethene backbone N3−C4−C5−N4 of a radical cation, as already calculated in the previous complexes. Moreover, the strong shortening of the Mn1−C3 bond [1.923(11) Å] in **[5]^2+^** relative to 2.0440(18) Å in **[5]^+^**, is consistent with an enhanced π-backbonding from the Mn center into the more π-acidic, oxidized ligand **1^•+^**. It is of note that this example represents the shortest Mn−NHC bond reported in the literature, being comparable only with two Mn(I) and Mn(IV) complexes bearing pincer (1.9360(16)) Å [60] and tripodal (1.938(2)) Å [61] imidazol-2-ylidene ligands, respectively. From a magnetic point of view, both the triplet state (S = 1) and broken symmetry state (Ms = 0) are quasi-isoenergetic (i.e., within DFT error, a few tens of cm^−1^). This does not allow us to conclude what the spin ground state of **[5]**^2+^ is (S = 1 or S = 0), and what is expected to be observed by EPR.

As it turns out, no signal is detected by recording the EPR spectra of complex **[5]**(SbF_6_)_2_ at various temperatures (from 4.2 K to room temperature). DFT only predicted that the two single electrons are expected to be weakly magnetically coupled. The fact that no EPR signal is observed could be due to a relatively high zero-field splitting constant of the EPR spectrum of the S = 1 (typically D > 1 cm^−1^), possibly associated with very short relaxation times. These features would lead either to low-field (X-band) EPR silent S = 1 system (D > 1cm^−1^) [62], or to a very broad EPR signal (very short relaxation times) [63], which is lower in intensity than the detection limit of our EPR spectrometer.

## 3. Materials and Methods

All manipulations were performed under an inert atmosphere of dry nitrogen by using standard vacuum line and Schlenk tube techniques. Glassware was dried at 120 °C in an oven, for at least three hours. THF, diethyl ether, pentane, toluene, and CH_2_Cl_2_ were dried using an innovative technology solvent purification system. 

Imidazolium triflate IMes^(NMe^^2)^^2^·HOTf (**1**·HOTf) [36], [RhCl(CO)_2_(IMes^(NMe^^2)^^2^)] [36], and [CpFe(CO)_2_I] [64] were prepared according to procedures in the literature. Following one such procedure, 1,1′-diacetylferrocenium tetrafluoroborate was prepared through the oxidation of 1,1′-diacetylferrocene by benzoquinone/HBF_4_∙OEt_2_ [65]. CuCl was purified by dissolution in the minimum of concentrated HCl, followed by precipitation by adding distilled water, and filtration through a fritted funnel. The off-white powder was dried carefully and stored in a glovebox. Technical quality [CpMn(CO)_3_] was purified by the crystallization from hexane, as described earlier [66]. The 0.5 M solution of KHMDS in toluene was prepared by weighing 2.0 g of solid KHMDS (stored in a glovebox) in a volumetric flask, and adding toluene to complete to 20 mL. AgSbF_6_ (Aldrich) was stored in the glovebox. All other reagents were commercially available, and used as received.

NMR spectra were recorded on Bruker AV300 or AV400 spectrometers. Chemical shifts are reported in ppm (δ) compared to TMS (^1^H and ^13^C), using the residual peak of deuterated solvent as internal standard [67]. Solution IR spectra were recorded in 0.1 mm CaF_2_ cells using a PerkinElmer Frontier FT-IR spectrometer, and given in cm^−1^ with relative intensities in parentheses. UV–vis spectra were recorded on a Perkin Elmer Lambda 950 UV/Vis/NIR spectrometer in 1 cm quartz cell under nitrogen atmosphere (CH_2_Cl_2_, 10^−4^ M sample concentration). Elemental analyses were performed by the microanalytical service of the LCC, and mass spectra (ESI mode) by the mass spectrometry service of the “Institut de Chimie de Toulouse”. 

X-band EPR data were recorded using Elexsys ESP 500 and EMX Brüker EPR spectrometers. Typical settings: 2 mW microwave power and 0.1 mT modulation amplitude. For all species, spectra recorded with lower modulation amplitudes do not reveal more lines or different patterns (data not shown). Landé factors were measured by comparison with a reference DPPH sample (g = 2.0036). The numerical simulation of EPR spectra was performed using EasySpin software [68].

**Chloro-(1,3-dimesityl-4,5-bis(dimethylamino)imidazol-2-ylidene)copper(I) (2**).

A solution of KHMDS (0.5 M in toluene, 610 µL, 0.31 mmol, 1.1 equiv) was added dropwise to a solution of **1**·HOTf (150 mg, 0.278 mmol) in THF (15 mL), at 0 °C. After 10 min, CuCl (55 mg, 0.56 mmol, 2 equiv.) was added as a solid, followed by NaCl (97.3 mg, 1.66 mmol, 6 equiv.), and the solution was stirred at room temperature for 1.5 h. After the evaporation of THF, the crude product was dissolved in toluene, and filtered over Celite. The toluene was evaporated, and the resulting brownish solid was washed with pentane, to afford complex **2** (133 mg, 96%) as a white powder.

^1^H NMR (400 MHz, CDCl_3_): δ = 6.96 (s, 4H, C*H*_Mes_), 2.58 (s, 12H, N−C*H*_3_), 2.33 (s, 6H, C*H*_3 para_), 2.12 (s, 12H, C*H*_3 ortho_).

^13^C{^1^H} NMR (101 MHz, CDCl_3_): δ = 171.8 (N_2_*C−*Cu), 138.9, 135.2, 134.3, 133.9 (*C*_Ar_), 129.5 (*C*H_Mes_), 43.3 (N−*C*H_3_), 21.3 (*C*H_3 para_), 18.3 (*C*H_3 ortho_).

MS (ESI, positive mode): *m/z* (%): 494 (100) [M–Cl + MeCN]^+^.

HRMS (ESI): *m/z* calcd. for C_27_H_37_N_5_^63^Cu^+^: 494.2345, found 494.2354, ε_r_ = 1.8 ppm.

**Generation and EPR characterization of** **[2]** **^+.^**

Solid 1,1′-diacetylferrocenium tetrafluoroborate (4.2 mg, 12 μmol, 1.0 equiv.) was added to a solution of complex **2** (5.8 mg, 12 μmol, 1.0 equiv.) in CH_2_Cl_2_ (1 mL) at –40 °C. After 5 min, 50 μL of the reaction mixture was syringed in an EPR tube at room temperature, which was completed with 50 μL of toluene. The EPR spectrum was immediately recorded. Compound **[2]^+^** was stable for at least 3 h at room temperature.

**Generation and EPR characterization of** **[3]** **^+.^**

Solid 1,1′-diacetylferricinium tetrafluoroborate (4.3 mg, 12 μmol, 1.0 equiv.) was added to a solution of complex **3** (7.0 mg, 12 μmol, 1.0 equiv.) in CH_2_Cl_2_ (1 mL), at −20 °C. The solution became violet. After 5 min, 50 μL of the reaction mixture was syringed in an EPR tube at room temperature, which was completed with 50 μL of toluene. The EPR spectrum was immediately recorded.

**(η^5^-Cyclopentadienyl)-Dicarbonyl-(1,3-Dimesityl-4,5-Bis(Dimethylamino)Imidazol-2-ylidene)iron (II) Tetrafluoroborate** **[4]** **(BF_4_).**

A solution of KHMDS (0.5 M in toluene, 380 µL, 0.19 mmol, 1.02 equiv.) was added dropwise to a solution of **1**·HOTf (100 mg, 0.185 mmol) in toluene (5 mL), at 0 °C. After 30 min, the toluene was evaporated, in order to remove HMDS, and the crude product was dissolved anew in 5 mL toluene, and filtered over Celite under nitrogen. The toluene was evaporated, and the carbene was dissolved in dry THF (5 mL). CpFe(CO)_2_I (61.8 mg, 0.2 mmol, 1.08 equiv.) was added as a solid in the dark (under visible light irradiation, a carbonyl ligand can be removed and replaced by the iodine counter-anion). The solution was stirred for 1 h at room temperature, and THF was evaporated (always in the absence of light). The solid was taken up in dichloromethane (5 mL), degassed water (5 mL) and NaBF_4_ (61.5 mg, 0.56 mmol, 3 equiv.) were added, and the reaction mixture was stirred for 30 min. The organic phase was isolated, dried over Na_2_SO_4_, and evaporated. The yellow solid was eventually washed with ether, and crystallized by the slow diffusion of a dichloromethane solution in diethyl ether, to afford **[4]**(BF_4_) (67 mg, 54%) as yellow crystals, some of which were suitable for X-ray diffraction.

^1^H NMR (400 MHz, CDCl_3_): δ = 7.07 (s, 4H, C*H*_Mes_), 4.73 (s, 5H, C*H*_Cp_), 2.54 (s, 12H, NC*H*_3_), 2.39 (s, 6H, C*H*_3 para_), 2.09 (s, 12H, C*H*_3 ortho_).

^13^C{^1^H} NMR (101 MHz, CDCl_3_): δ = 209.8 (Fe−*C*O), 162.7 (N_2_*C−*Fe), 140.6, 139.7, 136.3, 134.6 (*C*_Ar_), 130.0 (*C*H_Mes_), 86.3 (*C*H_Cp_), 43.5 (N−*C*H_3_), 21.3 (*C*H_3 para_), 18.7(*C*H_3 ortho_).

IR (CH_2_Cl_2_) ν_CO_ 2045 (s), 2001 (s) cm^−1^.

MS (ESI, positive mode): *m/z* (%): 362 (100) [M − BF_4_]^+^

HRMS (ESI): *m/z* calcd. for C_32_H_39_N_4_O_2_^56^Fe^+^: 567.2422, found 567.2432, ε_r_ = 1.8 ppm.

**Generation and EPR characterization of** **[4]** **^2+^**

Solid 1,1′-diacetylferrocenium tetrafluoroborate (4.2 mg, 12 μmol, 1.0 equiv.) was added to a solution of complex **[4]**(BF_4_) (7.8 mg, 12 μmol, 1.0 equiv.) in CH_2_Cl_2_ (1 mL), at –20 °C. After 5 min, 50 μL of the reaction mixture was syringed in an EPR tube at room temperature, which was completed with 50 μL of toluene. The EPR spectrum was immediately recorded. Compound **[4]^2+^** was stable for at least 3 h at room temperature.

IR (CH_2_Cl_2_): ν_CO_ 2054 (s), 2015 (s) cm^−1^.

**(η^5^-Cyclopentadienyl)-Dicarbonyl-(1,3-Dimesityl-4,5-Bis(Dimethylamino)Imidazol-2-Ylidene)Manganese (I) (5).**

To a suspension of **1**·HOTf (216 mg, 0.4 mmol) in toluene (30 mL), a solution of KHMDS (0.9 mL of 0.5 M, 0.45 mmol, 1.1 equiv.) was added, and the resulting, slightly yellow solution was sonicated for 5 min, and then stirred at RT for 15 min. CpMn(CO)_3_ (82 mg, 0.4 mmol, 1 equiv.) was added, and the reaction mixture was irradiated with UV light (370–380 nm), using a custom LED-based reactor (*ca.* 15W power), until ν_CO_ bands of the product at the measurements of 1904 and 1831 cm^–1^ ceased to increase (*ca.* 1h). The toluene was evaporated under vacuum, and the residue was purified by column chromatography on silica (1 × 6 cm). A slightly yellow band containing traces of cymantrene was eluted with 10:1 hexane/toluene mixture, followed by yellow–orange band of the target product. The solution was evaporated under vacuum, and the crude product was crystallized from ether/hexane mixture at –20 °C, to afford **5** (195 mg, 86%) as yellow crystals. Single crystals, suitable for X-ray diffraction, were obtained from concentrated solution in toluene at room temperature.

^1^H NMR (400 MHz, C_6_D_6_): δ = 6.92 (s, 4H, C*H*_Mes_), 4.02 (s, H, C*H*_Cp_), 2.23(s, 12H, NC*H*_3_), 2.20 (s, 18H, C*H*_3 Mes_).

^13^C{^1^H} NMR (101 MHz, C_6_D_6_): δ = 234.7 (Mn−*C*O), 198.0 (N_2_*C*−Mn), 138.1, 137.2, 137.1, 136.5 (*C*_Ar_), 129.4 (*C*H_Mes_), 81.8 (*C*H _Cp_), 43.4 (N−*C*H_3_), 21.2 (*C*H_3 para_), 19.3 (*C*H_3 ortho_).

IR (CH_2_Cl_2_): ν_CO_ 1904 (s), 1830 (s) cm^−1^.

UV–vis (CH_2_Cl_2_): λ 387 nm (ε 1400 M^−1^ cm^−1^).

Elemental analysis: *calcd* (%) for C_32_H_39_MnN_4_O_2_ (MW = 566.6): C 67.83, H 6.94, N 9.89; *found*: C 68.15, H 7.20, N 9.91.

**(η^5^-Cyclopentadienyl)-Dicarbonyl-(1,3-dimesityl-4,5-Bis(Dimethylamino)Imidazol-2-Ylidene)Manganese (II) Hexafluoroantimonate (** **[5]** **(SbF_6_)).**

To a stirred yellow solution of complex **5** (142 mg, 0.25 mmol) in CH_2_Cl_2_ (5 mL), solid AgSbF_6_ (86 mg, 0.25 mmol, 1 equiv.) was added, in one portion, at −20 °C. The color of the reaction turned green–brown, and the precipitate of metallic silver gradually appeared. The reaction mixture was warmed to room temperature, and stirred for 15 min. IR analysis of the aliquot shows the quantitative conversion of the starting material, and the appearance of new bands at ν_CO_ 2027 and 1950 cm^−1^, belonging to the oxidation product **[5]**(SbF_6_). The solvent was evaporated under vacuum; the residue was again dissolved in CH_2_Cl_2_ (2 mL), and filtered through Celite. Diethyl ether (20 mL) was added dropwise to the resulting green–brown solution under vigorous stirring, to induce the precipitation of the product. The supernatant was removed by decantation, and the precipitate was washed with additional Et_2_O (5 mL), and dried under vacuum to give **[5]**(SbF_6_) (182 mg, 91%) as black microcrystalline powder. Single crystals, suitable for X-ray diffraction, were obtained by vapor diffusion of ether into a concentrated solution of **[5]**(SbF_6_) in dichloromethane.

^1^H NMR (400 MHz, CD_2_Cl_2_, 25 °C): δ 14.09 (5H, w_½_ = 1300 Hz, Mn−Cp), 8.13 (4H, w_½_ = 70 Hz, CH_Mes_), 6.67 (6H, w_½_ = 300 Hz, Me), 5.96 (12H, w_½_ = 150 Hz, Me), 2.47 (12H, w_½_ = 180 Hz, Me).

IR (CH_2_Cl_2_): ν_CO_ 2027 (s), 1950 (s), ν_C=C_ 1665−1600 (m br) cm^−1^.

UV–vis (CH_2_Cl_2_): λ_1_ 434 (ε 1200 M^−1^ cm^−1^), λ_1_ 662 nm (ε 550 M^−1^ cm^−1^).

Elemental analysis: *calcd* (%) for C_32_H_39_F_6_MnN_4_O_2_Sb (MW = 802.4): C 47.90, H 4.90, N 6.98; *found*: C 48.17, H 5.09, N 6.64.

**(η^5^-Cyclopentadienyl)-Dicarbonyl-(1,3-Dimesityl-4,5-Bis(Dimethylamino)Imidazol-2-Ylidene)Manganese (II) (bis)Hexafluoroantimonate (** **[5]** **(SbF_6_)_2_).**

To a stirred yellow solution of complex **[5]**(SbF_6_) (105 mg, 0.13 mmol) in CH_2_Cl_2_ (5 mL), solid AgSbF_6_ (45 mg, 0.13 mmol, 1 equiv.) was added, in one portion, at −20 °C. The color of the reaction mixture immediately changed to deep blue, and black precipitate appeared. The reaction mixture was slowly warmed to room temperature, stirred for additional 30 min, and the volatiles were removed under vacuum. The residue was repeatedly extracted with CH_2_Cl_2_ under sonication (6 × 5 mL), combined dark blue solutions were filtered through Celite, and Et_2_O (25 mL) was added dropwise under vigorous stirring, to induce the precipitation of the product. The supernatant was removed by decantation, and the precipitate was washed with ether (10 mL) and dried under vacuum to give **[5]**(SbF_6_)_2_ (108 mg, 80%) as deep violet powder. Single crystals, suitable for X-ray diffraction, were obtained by the layering of toluene on the solution of **[5]**(SbF_6_)_2_ in CH_2_Cl_2_ at −20 °C.

^1^H NMR (400 MHz, CD_2_Cl_2_, 25 °C): δ 8.60 (8H, w_½_ = 300 Hz, Mn−Cp + Me), 7.11 (4H, w_½_ = 35 Hz, CH_Mes_), 5.04 (3H, w_½_ = 25 Hz, Me), 2.73–2.05 (several overlapping signals of variable width, 18H, Me), 1.26 (3H, w_½_ = 13 Hz, Me), 0.85 (3H, w_½_ = 30 Hz, Me).

IR (CH_2_Cl_2_): ν_CO_ 2024 (s), 1968 (s), ν_C=C_ 1602 (s) cm^−1^.

UV–vis (CH_2_Cl_2_): λ_1_ 386 (ε 2400 M^−1^ cm^−1^), λ_2_ 590 nm (ε 8000 M^−1^ cm^−1^).

Elemental analysis: *calcd* (%) for C_32_H_39_F_12_MnN_4_O_2_Sb_2_×CH_2_Cl_2_ (MW = 1123.1): C 35.29, H 3.68, N 4.99; *found*: C 35.32, H 3.72, N 4.79.

**Electrochemical studies**

Cyclic voltametric measurements were carried out with a Autolab PGSTAT100 potentiostat, controlled by GPES 4.09 software. Experiments were performed under argon at room temperature, in a homemade airtight three-electrode cell. The reference electrode consisted of a saturated calomel electrode (SCE) separated from the solution by a glass frit. The counter electrode was a platinum wire of ca. 1 cm^2^ apparent surface. The working electrode was a Pt microdisk (0.5 mm diameter). The supporting electrolyte, [nBu_4_N](PF_6_) (99% electrochemical grade), was dried at 120 °C and stored under Ar. The CH_2_Cl_2_ solutions of **2**, **3**, **[4]**(BF_4_), and **5** used for the electrochemical studies were typically 10^−3^ M in complex, and 0.1 M in supporting electrolyte. Before each measurement, the solutions were degassed by bubbling Ar, and the working electrode was polished with a polishing machine. Potentials are referenced to the SCE, and were calibrated using the Fc/Fc^+^ couple, by adding ferrocene (10^−3^ M) at the end of the experiments. Under the experimental conditions employed in this work, the half-wave potential (E_1/2_) of the Fc^+^/Fc couple in CH_2_Cl_2_ occurs at E_1/2_ = +0.46 V vs. SCE. Cyclic voltametric studies include the study of the compounds in oxidation, and in reduction at various scan rates (from 50 mV/s to 10 V/s).

**X-ray diffraction analyses.** X-ray diffraction data were collected on a Bruker SMART / APEX II diffractometer (**[4]**(BF_4_)), an Oxford diffraction Gemini diffractometer (**5**), an Xcalibur Gemini Ultra diffractometer (**[5]**(SbF_6_)), or on a Bruker D8/APEX II/Incoatec Mo IµS Microsource diffractometer (**[5]**(SbF_6_)_2_) using, in most cases, MoKα radiation (λ = 0.71073 Å, graphite monochromator), besides complex **[5]**(SbF_6_), for which CuKα radiation (λ = 1.54184 Å, graphite monochromator) was used. Crystal and structure refinement details are given in the Table S1. All calculations were performed on a PC compatible computer using the WinGX system [69]. The structures were solved using the SIR92 program [70], which revealed in each instance the position of most of the non-hydrogen atoms. All the remaining non-hydrogen atoms were located by the usual combination of full matrix least-squares refinement, and difference electron density syntheses using the SHELX program [71]. Atomic scattering factors were taken from the usual tabulations. Anomalous dispersion terms for the Mn and Fe atoms were included in Fc. All non-hydrogen atoms were allowed to vibrate anisotropically. The hydrogen atoms were set in idealized positions (R_3_CH, C–H = 0.96 Å; R_2_CH_2_, C–H = 0.97 Å; RCH_3_, C–H = 0.98 Å; C(sp^2^)–H = 0.93 Å; U*_iso_* 1.2 or 1.5 times greater than the Ueq of the carbon atom to which the hydrogen atom is attached), and their positions refined as “riding” atoms. After completing the initial structure solution of complexes **[5]**(SbF_6_) and **[5]**(SbF_6_)_2_, it was found that ca. 10% and 37% of the total cell volume was filled with disordered solvent, accounting for 84 and 386 electrons per unit cell, respectively. These solvent molecules, likely to be Et_2_O in partial occupation in case of **[5]**(SbF_6_) and CH_2_Cl_2_/toluene for **[5]**(SbF_6_)_2_, could not be well-modelled in terms of atomic sites, and the SQUEEZE procedure [72] was, therefore, applied to the data to remove this contribution. CCDC 2170556-2170559 contains the supplementary crystallographic data for the structures unveiled in this paper. These data can be obtained, free of charge, from the Cambridge Crystallographic Data Centre via www.ccdc.cam.ac.uk/data_request/cif.

## 4. Conclusions

Following a first study on the thiourea derivatives, we then conducted a complete exploration of the redox behavior of a series of transition-metal complexes supported by the NHC ligand IMes^(NMe^^2)^^2^ (**1**), whose carbenic heterocycle is substituted by two dimethylamino groups. The ability of ligand **1** to act as a redox-active ligand is shown through different spectroscopic, and even crystallographic, techniques on the oxidized forms of the complexes. In a similar behavior to the thiourea derivative, the two dimethylamino groups in **1** play a critical role in the stabilization of the mono-oxidized ligand [IMes^(NMe^^2)^^2^]**^•+^** (**1^•+^**) in the complexes, by allowing an efficient delocalization of the spin density over the diaminoethene backbone. Conversely, the bis-oxidized state of the ligand is not generated in these complexes. The oxidation process of the NHC ligand is shown to have a profound effect on its overall electronic donation, leading to a new redox-switchable NHC. An obvious limitation of this ligand is a relatively high oxidation potential, which would limit its implementation in organometallic catalysis. We are, therefore, considering further developments, aimed at improving both the accessibility and stability of the oxidized forms of the NHC ligands, by changing the nature and location of the redox-active moiety, the NHC platform, and/or the denticity of the NHC ligand. Investigations along those lines are currently being performed in our laboratories.

## Data Availability

Data is available in the Supplementary Material.

## Supplementary Materials

The following supporting information can be downloaded at: https://www.mdpi.com/article/10.3390/molecules27123776/s1: SI containing IR, UV-Vis, and NMR spectra, crystallographic information and the atomic coordinates of the calculated structures [73,74,75,76].

## Sample Availability

Samples of the compounds might be available from the authors.
