# Supplementary material for: Redox-Switchable Behavior of Transition-Metal Complexes Supported by Amino-Decorated N-Heterocyclic Carbenes
Source: Molecules. 2022 Jun 11;27(12):3776. doi: 10.3390/molecules27123776 (PMC9227367; doi:10.3390/molecules27123776)
Supplement: Supplementary file 1 [file molecules-27-03776-s001.zip › molecules-1765327-supplementary.pdf]

Redox-Switchable Behavior of Transition-Metal Complexes Supported by Amino-Decorated N-Heterocyclic Carbenes

Mirko Ruamps, Stéphanie Bastin, Lionel Rechignat, Alix Sournia-Saquet, Laure Vendier, Noël Lugan, Jean-Marie Mouesca, Dmitry A. Valyaev, Vincent Maurel,* Vincent César**

Supporting Information

Contents

Figure S1. Cyclic voltammogram of [CuCl(IMes)] S2

Figure S2. IR spectra of manganese NHC complexes **5**, [**5**](SbF_6) and [**5**](SbF_6)$_2$ S2

Figure S3. UV-Vis spectra of manganese NHC complexes **5**, [**5**](SbF_6) and [**5**](SbF_6)$_2$ S3

Table S2. Crystal data and structure refinement for [**4**](BF_4), **5**, [**5**](SbF_6) and [**5**](SbF_6)$_2$ S4

Figures S4-S11. NMR spectra of NHC complexes **2**, [**4**](BF_4), **5**, [**5**](SbF_6) and [**5**](SbF_6)$_2$ S5

Atomic coordinates of [2]$^+$, [3]$^+$, [4]$^{2+}$, [5]$^+$, and [5]$^{2+}$ S9

Figure S1. Cyclic voltammogram of complex [CuCl(IMes)]. Pt working electrode, 1 mM in CH_2Cl_2 with 0.1 M Bu_4NPF_6 as the supporting electrolyte, 200 mV.s^{-1} scan rate, potentials were calibrated against Fc/Fc^+ as internal standard with $E_{1/2}$ = +0.46 V *vs.* SCE

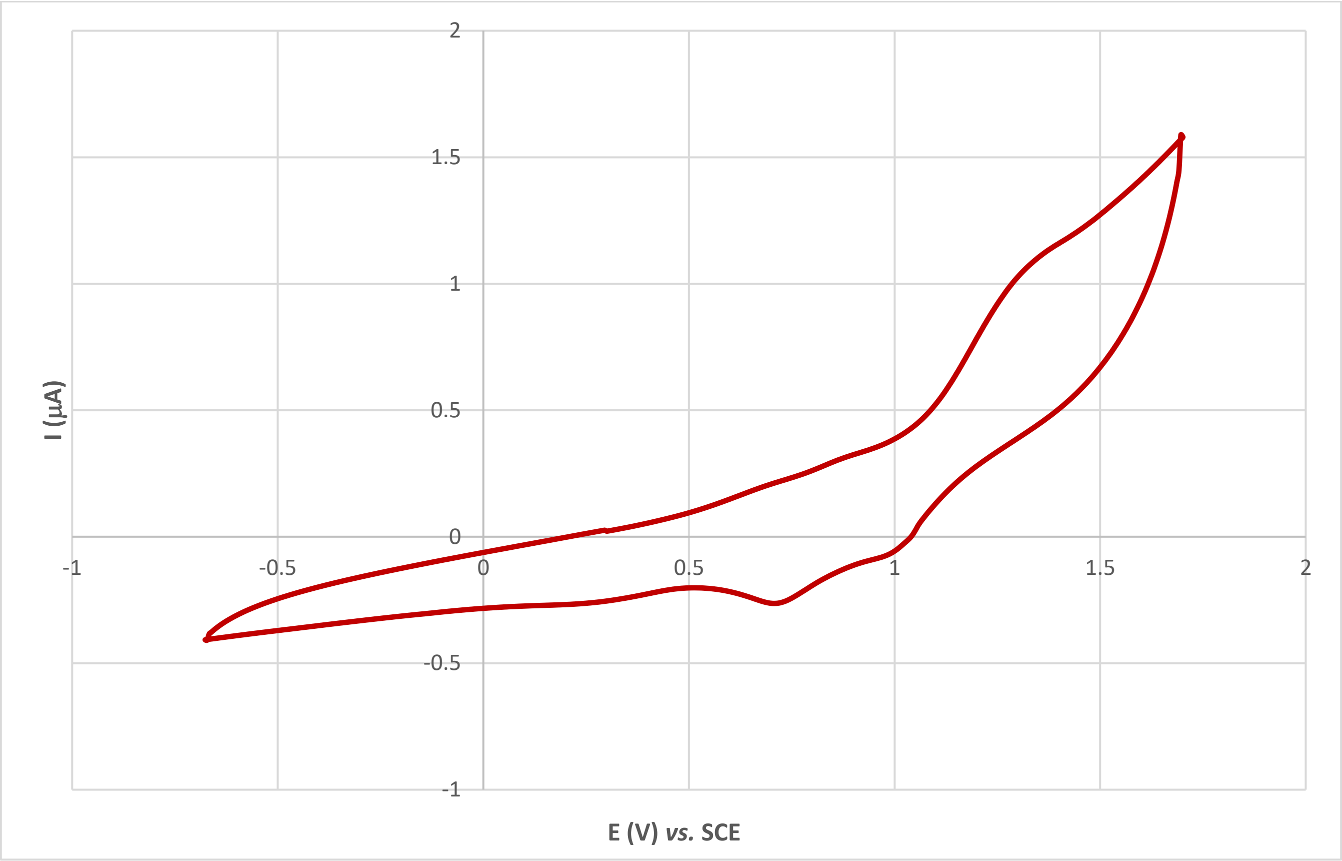

Figure S2. Normalized IR spectra of manganese NHC complexes **5**, [**5**](SbF_6) and [**5**](SbF_6)$_2$ in (CH_2Cl_2 solution, 2200-1500 cm^{-1} region).

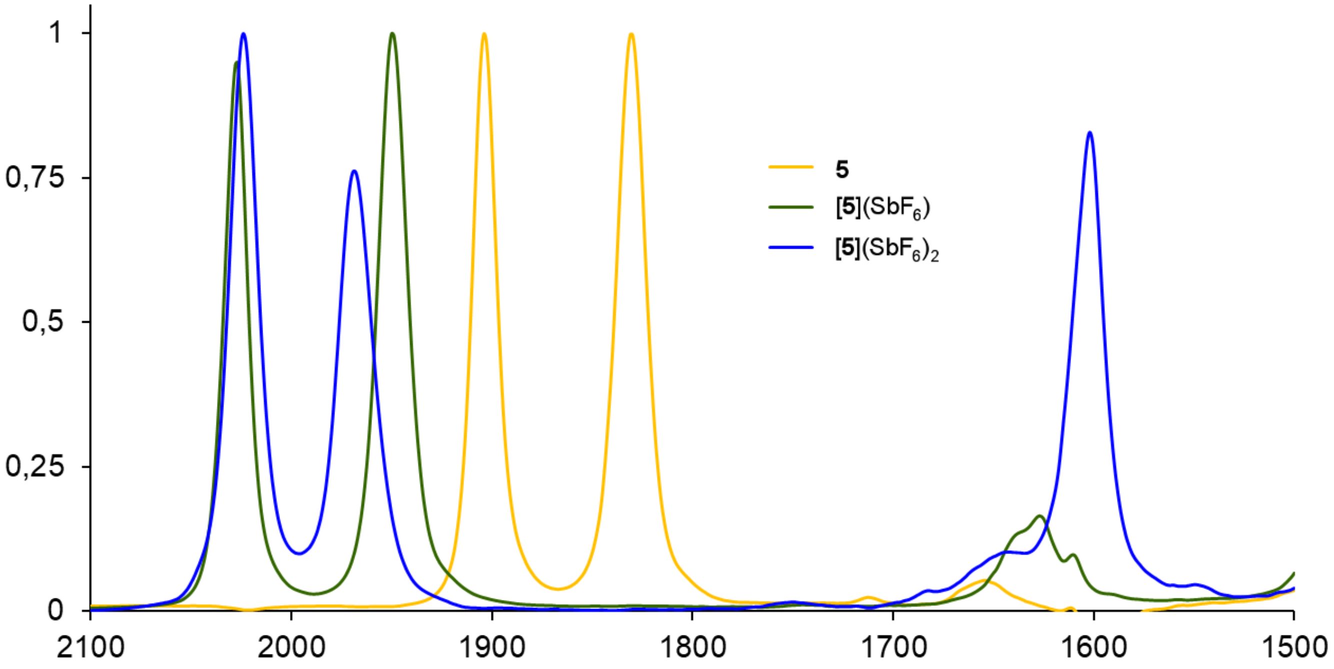

Figure S3. Normalized UV/Vis spectra of manganese NHC complexes **5**, [**5**](SbF_6) and [**5**](SbF_6)$_2$ (CH_2Cl_2 solution, 10^{-4} M level of concentrations).

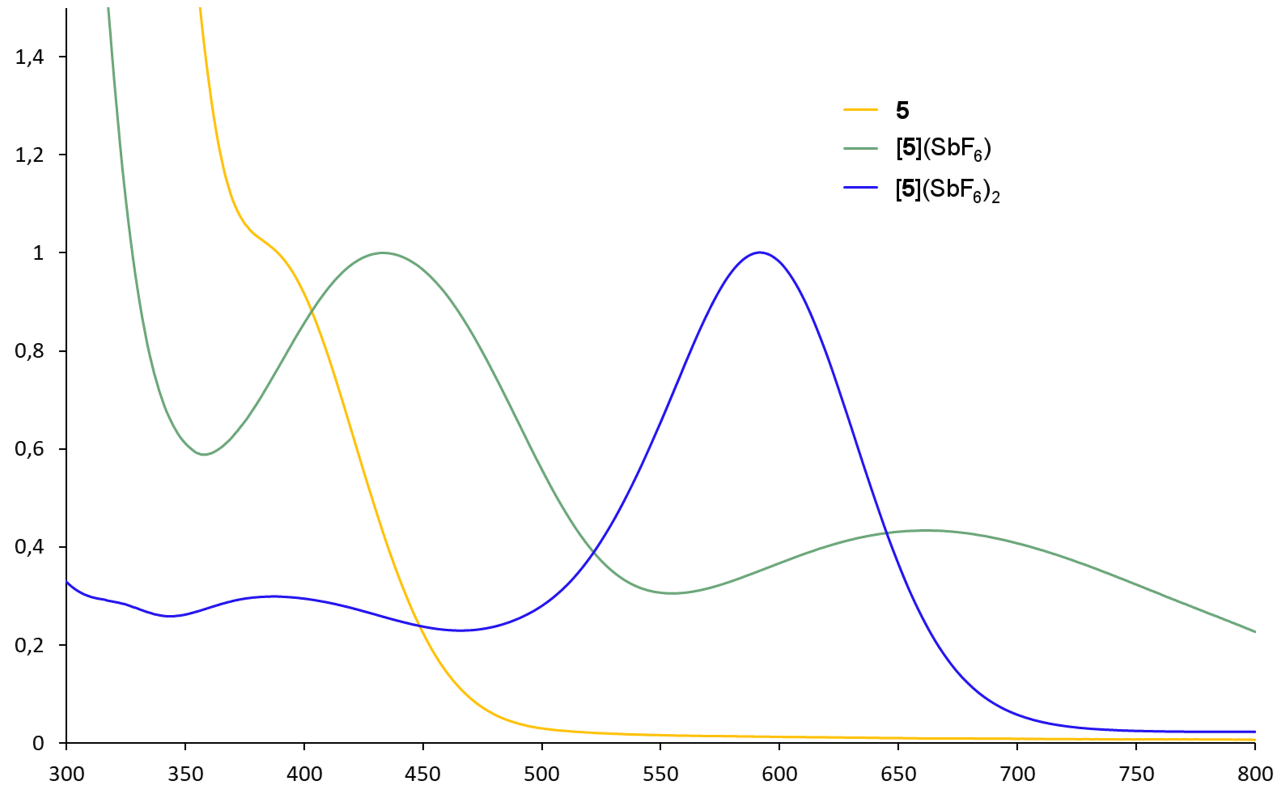

Table S1. Crystal data and structure refinement for NHC complexes [**4**](BF_4), **5**, [**5**](SbF_6) and [**5**](SbF_6)$_2$

Complex	[**4**](BF_4)	**5**	[**5**](SbF_6)	[**5**](SbF_6)$_2$
Empirical formula	$C_{12}H_{13}BF_4FeN_2O_2$	$C_{32}H_{39}MnN_4O_2$	$C_{32}H_{39}F_6MnN_4O_2Sb$	$C_{32}H_{39}F_{12}MnN_4O_2Sb_2$
M_r	654.33	566.61	802.36	1038.13
T/K	173	123	150	100
λ/Å	0.71073	0.71073	1.54184	0.71073
Crystal system	orthorhombic	monoclinic	monoclinic	triclinic
Space group (no.)	$P2_12_12_1$ (#19)	$P2_1/c$ (#14)	$P2_1/n$ (#14)	P1 bar (#2)
a/Å	9.1166(3)	16.9220(6)	12.95920(10)	8.8818(13)
b/Å	18.2281(5)	16.4516(3)	17.97800(10)	16.660(2)
c/Å	19.4675(5)	21.2589(6)	15.77180(10)	19.335(3)
α/°	90	90	90	97.327(4)
β/°	90	90.000(3)	97.8330(10)	101.687(4)
γ/°	90	90	90	99.991(4)
V/Å^3	3235.08(16)	5918.3(3)	3640.24(4)	2719.7(7)
Z	4	4	4	2
D_c/g.cm^{-3}	1.343	1.272	1.464	1.268
μ/mm^{-1}	0.524	0.481	9.262	1.281
F(000)	1368	2400	1620	1020
θ_{max}/°	26.4	26.4	71.4	26.4
Completeness to θ_{max} (%)	0.997	0.984	0.997	0.981
Index range, *hkl*	−11<h<11<br>−22<k<22<br>−24<l<24	−21<h<20<br>−20<k<20<br>−26<l<26	−15<h<15<br>−22<k<22<br>−19<l<19	−10<h<11<br>−20<k<20<br>−24<l<24
Reflections collected	6600	11898	7059	38907
Independent reflections	6172	10775	6497	10900
Parameters	407	724	425	491
GOF	1.03	1.03	1.04	1.11
R (all data)	0.0328	0.0392	0.027	0.1413
R [I>2σ(I)]	0.0289	0.0328	0.024	0.1191
Rw (all data)	0.0845	0.0845	0.063	0.26
R_w [I>2σ(I)]	0.0813	0.0803	0.0612	0.2484
$\Delta\rho_{max/min}$/e.Å^{-3}	−0.16/0.44	−0.38/0.30	−0.61/0.44	−2.20/1.38

NMR Spectra

Figure S4. ^{1}H NMR spectrum of **2** (400 MHz, $CDCl_3$)

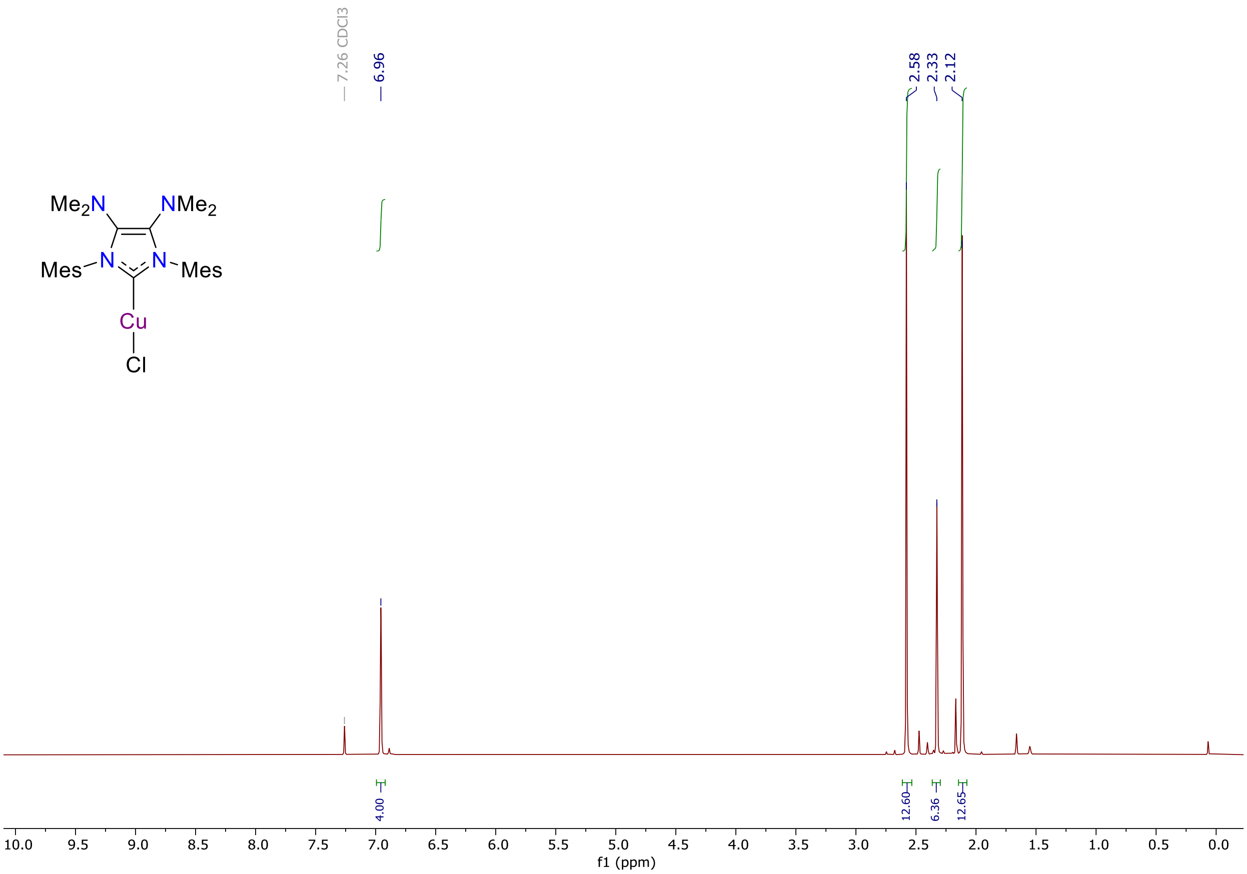

Figure S5. $^{13}C\{^1H\}$ NMR spectrum of **2** (100.6 MHz, $CDCl_3$)

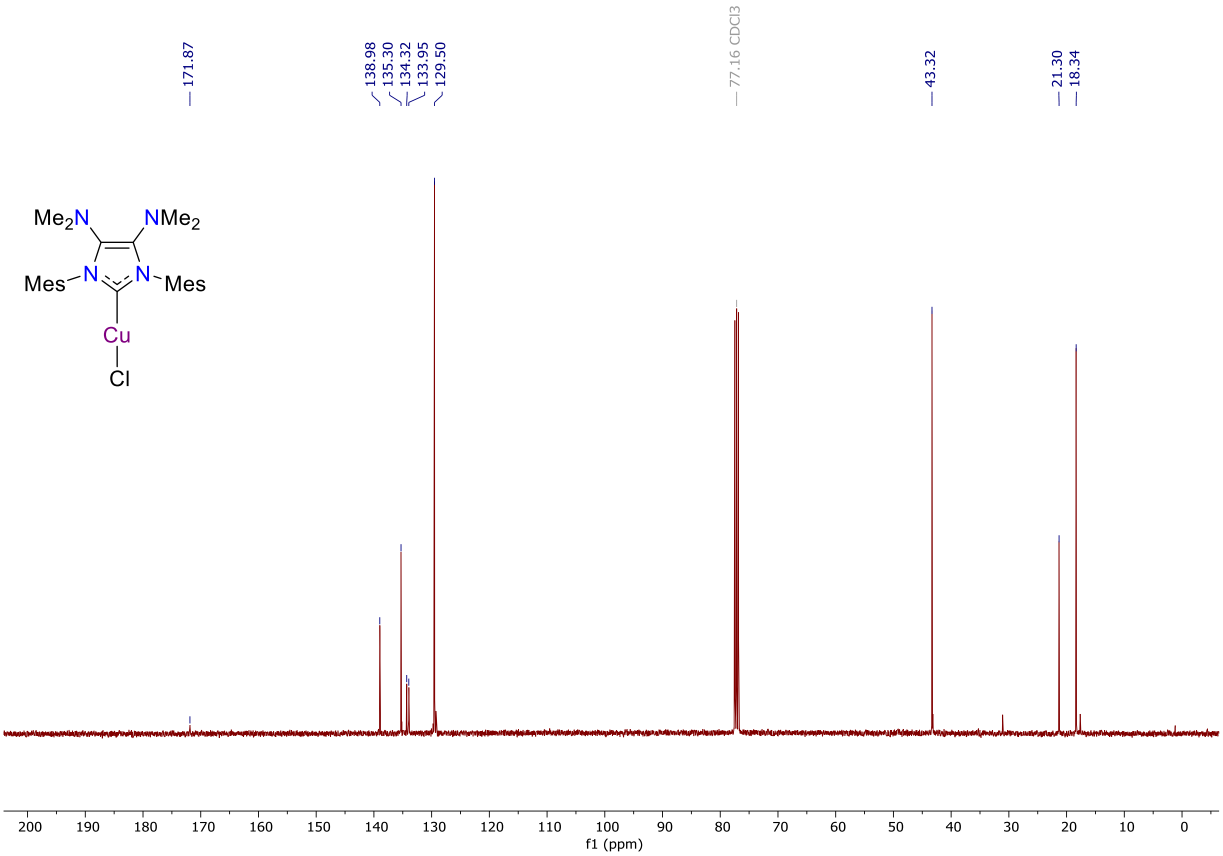

Figure S6. ^{1}H NMR spectrum of [**4**](BF_4) (400 MHz, $CDCl_3$)

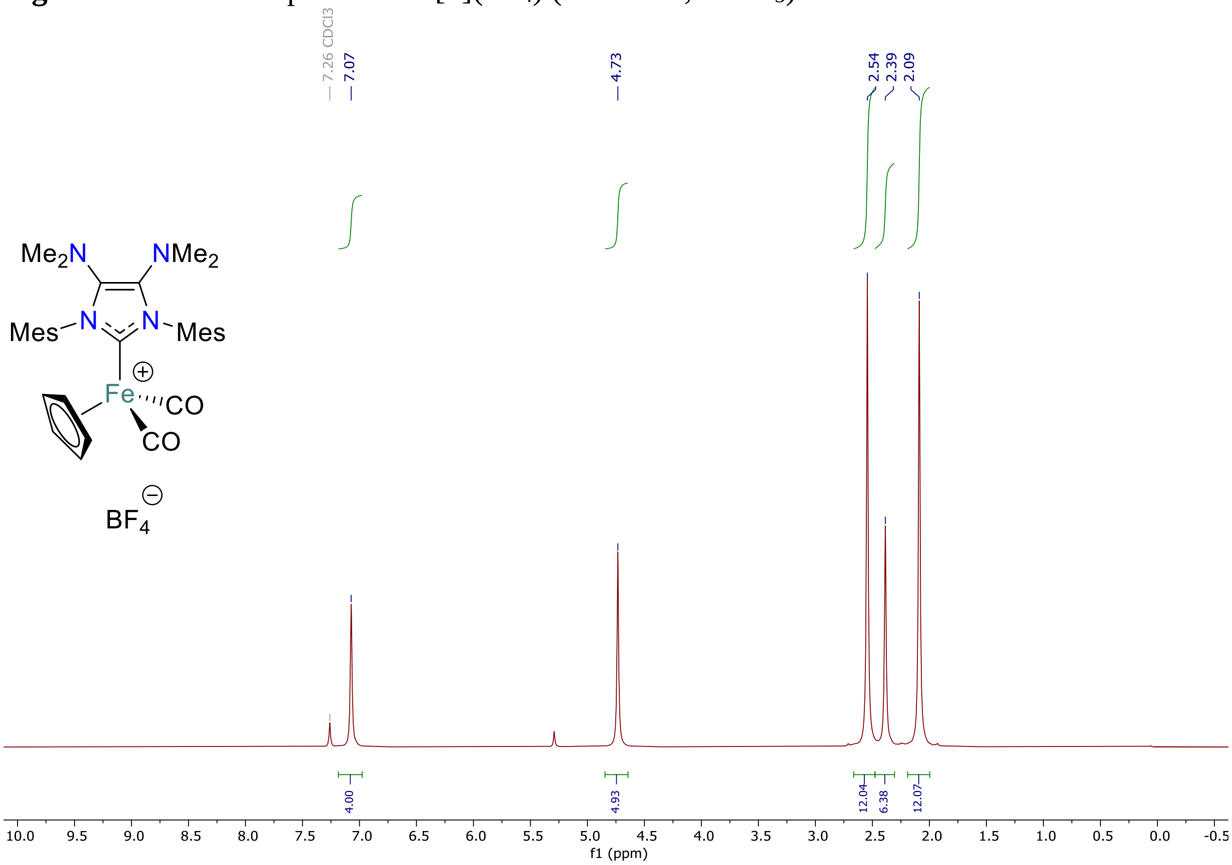

Figure S7. ^{13}C{^{1}H} NMR spectrum of [**4**](BF_4) (100.6 MHz, $CDCl_3$)

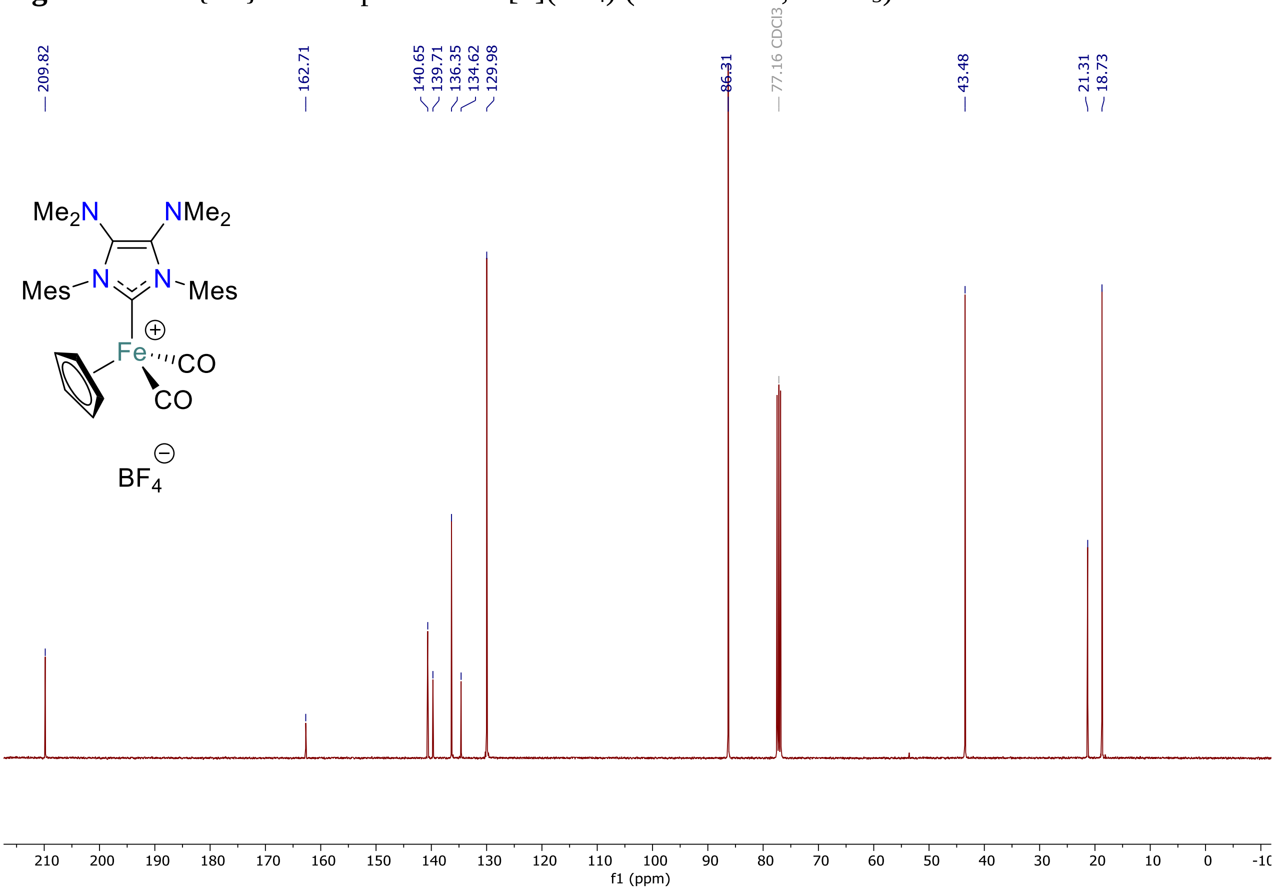

Figure S8. ^{1}H NMR spectrum of **5** (400 MHz, C_6D_6)

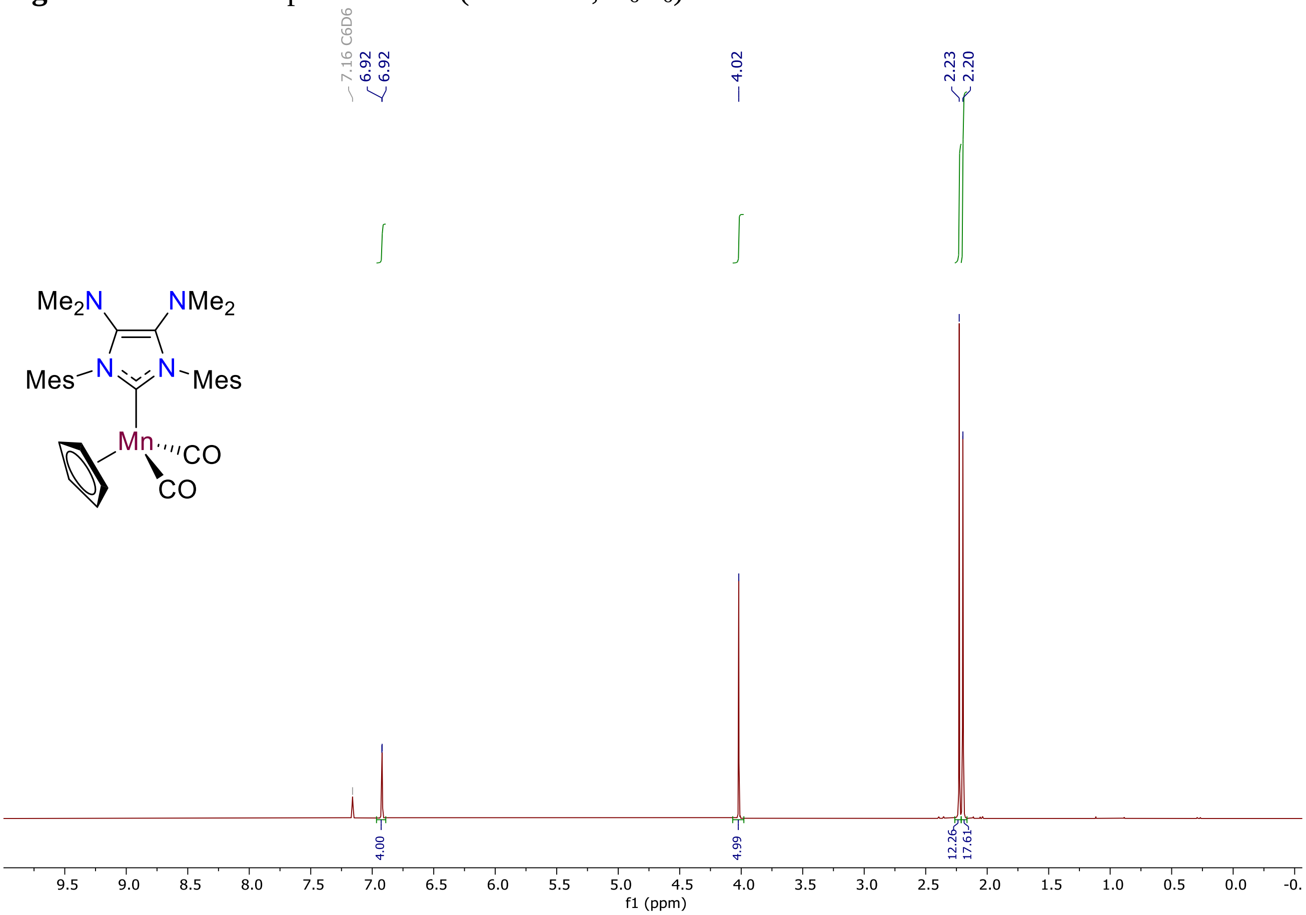

Figure S9. $^{13}C\{^{1}H\}$ spectrum (JMOD) of **5** (400 MHz, C_6D_6)

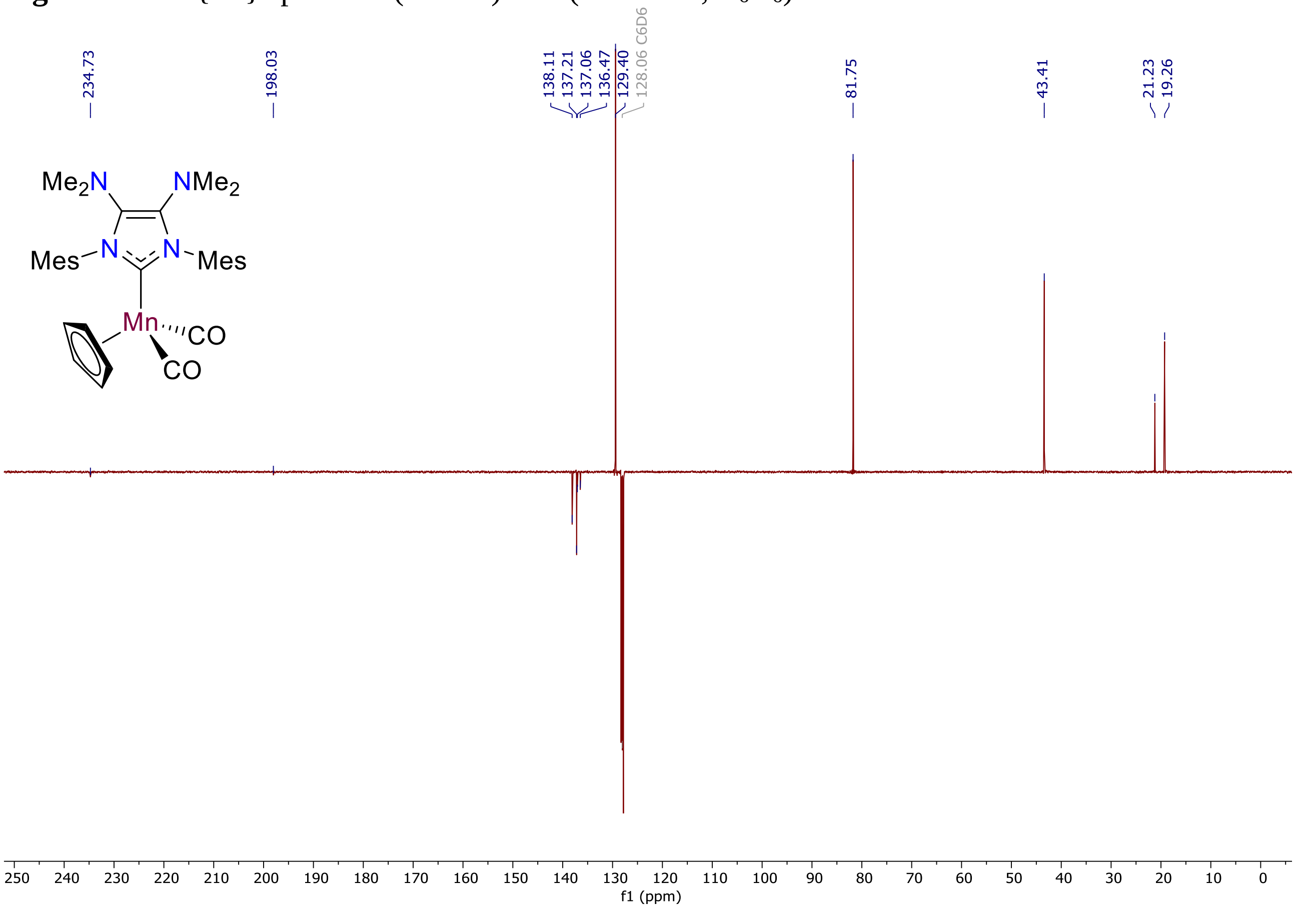

Figure S10. ^{1}H NMR spectrum of [**5**](SbF_6) (400 MHz, CD_2Cl_2), signals of Et_2O are marked with asterisk

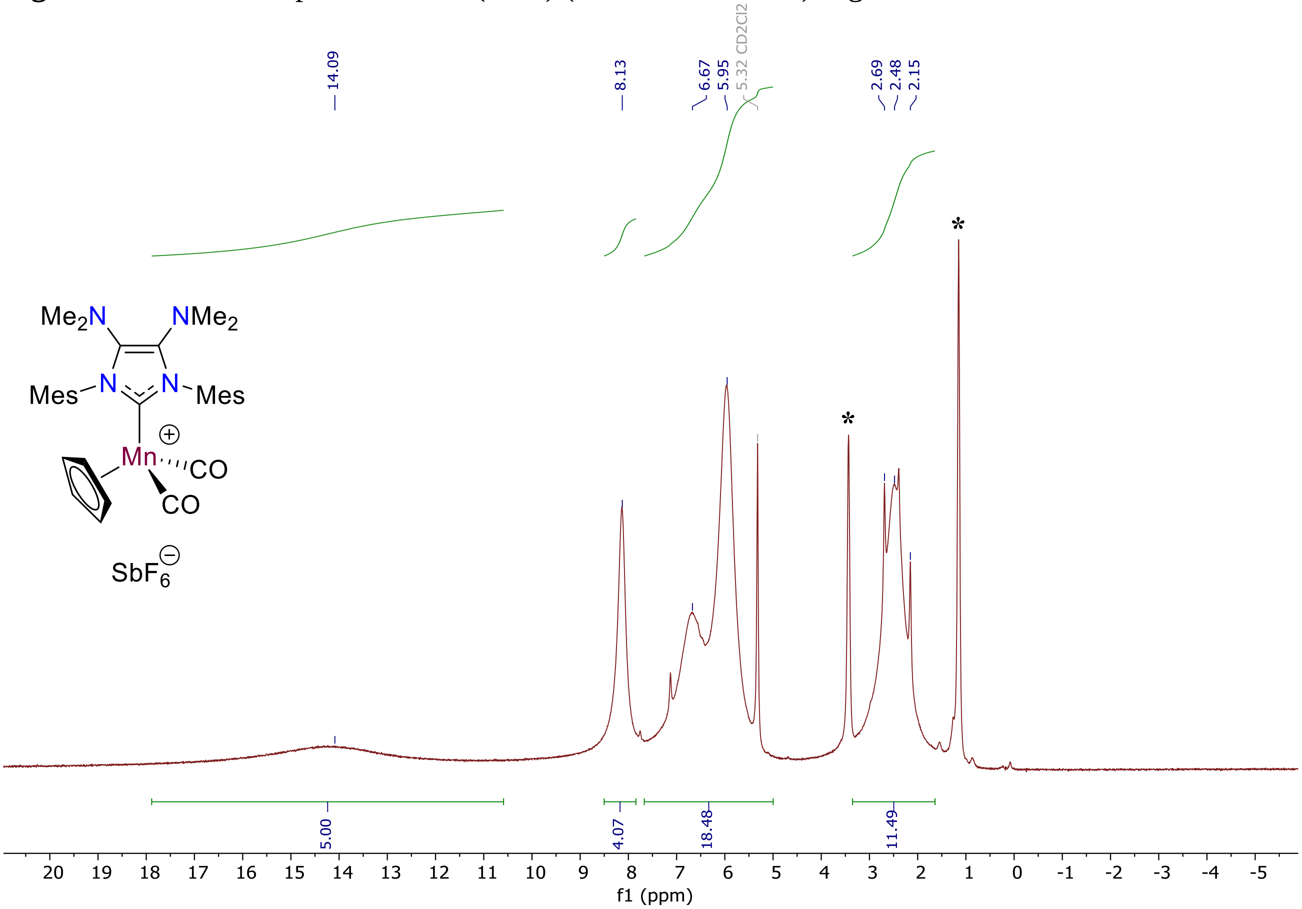

Figure S11. ^{1}H NMR spectrum of [**5**](SbF_6)$_2$ (400 MHz, CD_2Cl_2), signals of grease is marked with asterisk

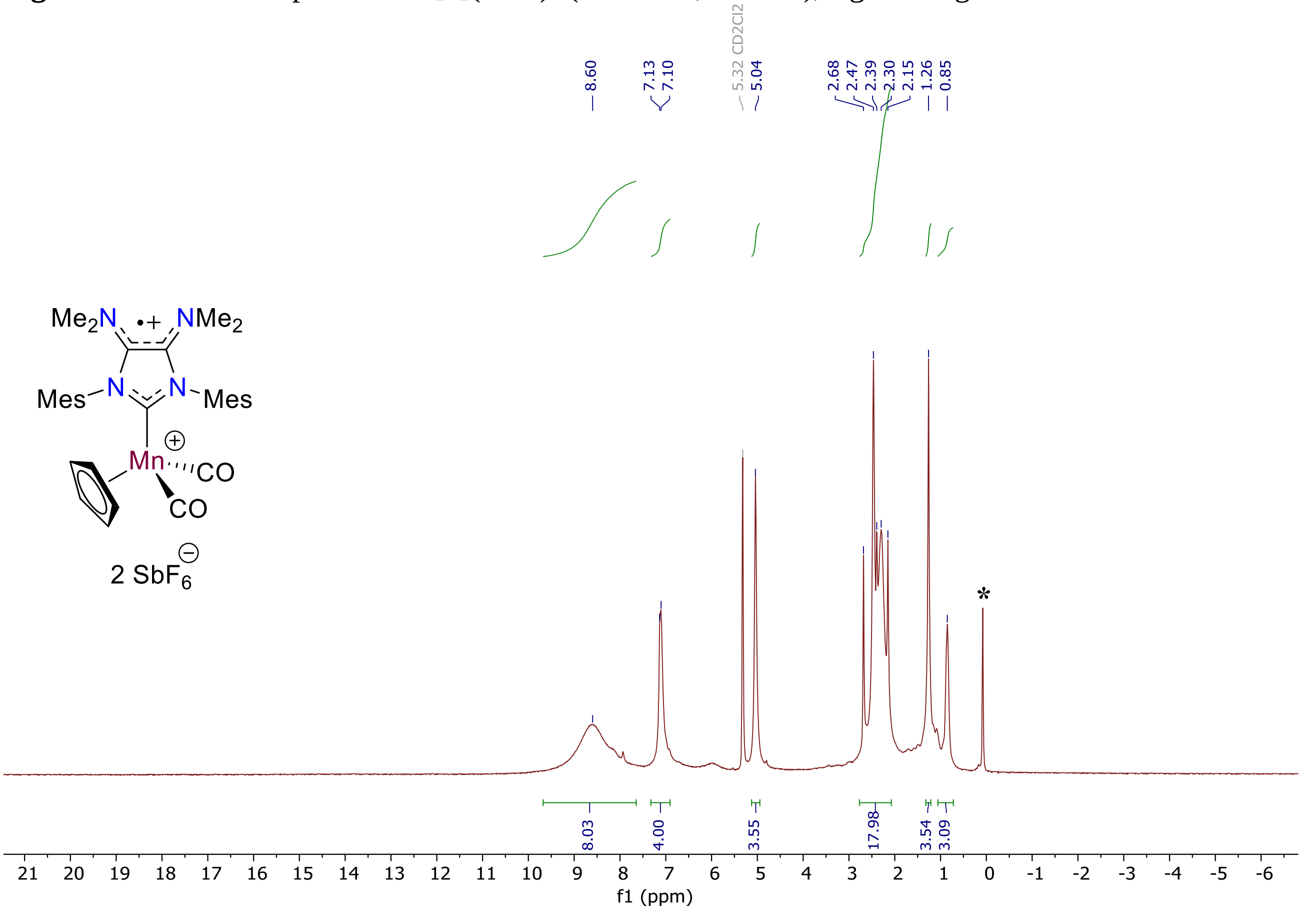

DFT-optimized geometries

All DFT calculations were performed with the ADF (Amsterdam Density Functional) code developed by E. J. Baerends and co-workers[1] using triple-zeta (TZ2P) basis sets (no frozen core) (relativistic ZORA/TZ2P for Rhodium). Geometry optimizations were performed *in vacuo* relying on the Generalized Gradient Approximation (GGA) VBP exchange-correlation (XC) potential (VWN + BP: Vosko, Wilk & Nusair[2] + corrective terms by Becke[3] for the exchange, and Perdew[4] for the correlation) with ADF grid precision 6 throughout.

[1] G. te Velde, E. J. Baerends, J. Comput. Phys. 1992, 99, 84–98.
[2] S. H. Vosko, L. Wilk, M. Nusair, Can. J. Phys. 1980, 58, 1200–1211.
[3] A. D. Becke, Phys. Rev. A 1988, 38, 3098–3100.
[4] J. P. Perdew, Phys. Rev. B 1986, 33, 8822–8824.

$[CuCl(IMes^{(NMe_2)2})]^+ = [2]^+$

Cu	3.900422	4.642614	11.055449
C	3.982565	3.073204	12.057146
C	4.774003	1.186874	13.071428
C	3.379379	1.301622	13.365282
C	5.597912	-0.204438	14.927148
H	6.512358	0.118227	15.448680
H	5.548225	-1.301127	14.969302
H	4.733403	0.221405	15.442415
C	6.850419	-0.160728	12.803148
H	6.724217	-0.001179	11.729689
H	6.999206	-1.234191	12.987015
H	7.746029	0.380267	13.144055
C	2.745096	-1.023175	13.866226
H	3.600012	-1.245370	13.222825
H	1.835043	-1.422640	13.392280
H	2.874313	-1.523238	14.835854
C	1.396797	0.821793	14.793358
H	0.481008	0.677291	14.200478
H	1.460832	1.867951	15.101757
H	1.335375	0.190498	15.691110
C	6.382494	2.641086	11.724858
C	7.274426	3.323089	12.570540
C	8.510041	3.701207	12.035575
H	9.209324	4.243081	12.674227
C	8.862588	3.431227	10.707898
C	7.931810	2.767955	9.899943
H	8.177202	2.568178	8.855810
C	6.681083	2.365413	10.379700
C	6.915146	3.688880	13.988495
H	7.722336	4.265587	14.452236
H	6.736561	2.804124	14.617119
H	6.003143	4.302048	14.020858
C	10.208120	3.840080	10.165742
H	10.945373	3.035913	10.309002
H	10.591713	4.730849	10.676932
H	10.159326	4.052121	9.091289
C	5.693554	1.696430	9.457631
H	4.878148	2.385945	9.192397
H	5.228658	0.808456	9.908816

H	6.182354	1.387410	8.527603
C	1.608330	3.016186	12.703121
C	1.269503	4.087735	13.544978
C	-0.029760	4.599686	13.448524
H	-0.308668	5.435657	14.091442
C	-0.968057	4.087027	12.547090
C	-0.575482	3.030664	11.714444
H	-1.283411	2.633488	10.985060
C	0.707380	2.478844	11.766034
C	2.259350	4.705066	14.499908
H	2.804822	3.953295	15.087771
H	1.752529	5.378978	15.198705
H	3.011867	5.294458	13.954980
C	-2.362519	4.652995	12.464956
H	-3.092115	3.961166	12.910747
H	-2.663890	4.812189	11.421704
H	-2.440981	5.608780	12.994584
C	1.105243	1.384976	10.807522
H	0.270541	1.132545	10.145121
H	1.409386	0.463308	11.324768
H	1.949913	1.699496	10.177676
N	5.078298	2.263702	12.244706
N	2.958931	2.481169	12.758906
N	5.649890	0.254625	13.534778
N	2.596638	0.424708	14.050497
Cl	3.807782	6.388941	9.933897

[RhCl(COD)(IMes$^{(NMe_2)_2}$)]$^+$ = [3]$^+$

Rh	3.909596	4.862127	10.937715
C	5.495943	5.694861	11.737335
O	6.341381	6.350589	12.143054
C	2.262823	4.458257	9.954440
O	1.352228	4.381450	9.265321
C	3.981625	3.140479	12.011865
C	4.770683	1.252901	13.056176
C	3.365807	1.309734	13.254769
C	5.548848	-0.171074	14.907564
H	6.459290	0.111744	15.459270
H	5.467757	-1.267253	14.927009
H	4.685005	0.267128	15.414158
C	6.832141	-0.129486	12.801481
H	6.728671	0.068691	11.731601
H	6.928628	-1.215175	12.949613
H	7.749674	0.355499	13.168720
C	2.762374	-1.031144	13.720435
H	3.614069	-1.228079	13.064120
H	1.857867	-1.457079	13.258108
H	2.919003	-1.534793	14.684987
C	1.393957	0.778613	14.688325
H	0.461600	0.608398	14.128219
H	1.440460	1.827669	14.991540
H	1.376577	0.152500	15.592548
C	6.446628	2.735213	11.900778
C	7.319125	3.250564	12.874740
C	8.625801	3.559517	12.477483
H	9.311429	3.972075	13.219112

C	9.069826	3.373698	11.164834
C	8.161027	2.864881	10.226898
H	8.484290	2.718705	9.194887
C	6.845416	2.538500	10.564497
C	6.886190	3.504158	14.297342
H	7.602190	4.161064	14.802829
H	6.826721	2.574096	14.881828
H	5.898322	3.981822	14.345902
C	10.474524	3.735001	10.756020
H	10.942889	2.924797	10.181859
H	11.105705	3.950741	11.625182
H	10.472855	4.626550	10.112539
C	5.902020	2.011889	9.514580
H	5.157890	2.778240	9.248250
H	5.344621	1.128514	9.857364
H	6.447456	1.738590	8.605201
C	1.546135	2.887437	12.522621
C	1.101785	3.962031	13.316281
C	-0.248091	4.313472	13.233093
H	-0.607402	5.144288	13.842499
C	-1.146435	3.638437	12.396191
C	-0.656965	2.582750	11.620202
H	-1.335456	2.055620	10.947716
C	0.684763	2.186219	11.660563
C	2.031243	4.731087	14.218272
H	2.656372	4.070924	14.836392
H	1.466462	5.389174	14.886871
H	2.714409	5.355114	13.621914
C	-2.588757	4.065901	12.309274
H	-3.221570	3.271730	11.897052
H	-2.689998	4.944094	11.654576
H	-2.982998	4.349004	13.293165
C	1.164368	1.064667	10.772954
H	0.433865	0.865600	9.981410
H	1.303765	0.127421	11.331897
H	2.123569	1.303284	10.294063
N	5.099111	2.373455	12.294071
N	2.931040	2.469585	12.614210
N	5.643294	0.318490	13.530254
N	2.578732	0.409651	13.910318
Cl	3.834763	6.854779	9.690506

$[FeCp(CO)_2(IMes^{(NMe_2)_2})]^{2+} = [4]^{2+}$

Fe	11.650658	4.347856	6.015645
C	10.485754	3.228739	5.283479
C	10.791642	5.731090	5.301255
C	9.885196	3.847308	9.830887
C	9.977707	5.269858	9.791691
C	9.900098	3.476751	12.263819
H	10.487689	4.397564	12.266036
H	10.503063	2.670318	12.706030
H	9.000545	3.603829	12.880637
C	8.930997	1.743201	10.817967
H	8.238735	1.654775	11.665976
H	9.686211	0.948251	10.901259
H	8.371725	1.618362	9.889944

C	10.025014	7.481309	10.975194
H	9.863026	7.677519	12.043592
H	9.517836	8.265932	10.395190
H	11.096384	7.510106	10.769291
C	8.189805	5.888867	11.364235
H	7.743884	4.955114	11.013870
H	7.504916	6.717092	11.130304
H	8.322768	5.857282	12.453746
C	13.624887	4.204538	6.873931
H	13.864671	4.260658	7.928854
C	13.323679	3.011379	6.152076
H	13.296946	2.002126	6.546402
C	13.101594	3.381851	4.785286
H	12.867256	2.703534	3.971042
C	13.247584	4.791020	4.676104
H	13.146618	5.371913	3.765054
C	13.571148	5.306973	5.976901
H	13.760099	6.347815	6.213244
C	10.410232	1.991762	8.218347
C	11.531415	1.235410	8.612940
C	11.561096	-0.116125	8.254347
H	12.427976	-0.713273	8.544370
C	10.515453	-0.727834	7.548421
C	9.408798	0.057430	7.207342
H	8.579768	-0.399171	6.664680
C	9.322042	1.415147	7.537046
C	12.658964	1.798870	9.444915
H	12.541203	1.517262	10.501882
H	12.717059	2.891686	9.402749
H	13.625857	1.391285	9.125936
C	10.574136	-2.190951	7.197165
H	9.832299	-2.454826	6.435932
H	10.374678	-2.808907	8.084948
H	11.567485	-2.474071	6.827231
C	8.060990	2.174806	7.202563
H	7.638790	1.822744	6.255152
H	8.218121	3.256046	7.121017
H	7.286915	2.012677	7.969005
C	10.827529	6.951713	8.135470
C	12.072418	7.566378	8.361593
C	12.227687	8.894080	7.944376
H	13.187007	9.385877	8.116761
C	11.189399	9.616680	7.345266
C	9.956771	8.972313	7.172995
H	9.130101	9.519147	6.716998
C	9.744357	7.646137	7.558934
C	13.208162	6.900376	9.102602
H	13.418881	7.432357	10.041614
H	14.140647	6.934699	8.524915
H	12.999710	5.856045	9.355166
C	11.390169	11.036831	6.888080
H	11.666683	11.060753	5.823384
H	12.192238	11.532619	7.446329
H	10.472855	11.627379	6.996154
C	8.384373	7.022011	7.363062
H	7.828062	7.552903	6.583349

H	7.780109	7.076745	8.281478
H	8.441325	5.965603	7.071453
C	10.816155	4.447482	7.814034
N	10.342850	3.398143	8.587003
N	10.608449	5.583331	8.582935
N	9.540421	3.079341	10.893632
N	9.471216	6.149390	10.690285
O	9.796314	2.557183	4.649799
O	10.283586	6.561407	4.685973

$[MnCp(CO)_2((IMes^{(NMe_2)_2})]^+ = [5]^+$

Mn	11.594456	4.337599	5.928137
C	11.406497	2.576236	5.593136
C	9.966949	4.519418	5.199679
C	9.747850	3.910667	9.755246
C	9.923400	5.293109	9.750350
C	9.774202	3.380886	12.146061
H	10.687973	3.983838	12.133391
H	9.991352	2.431121	12.657812
H	9.003625	3.906929	12.735958
C	8.162994	2.225292	10.701464
H	7.300658	2.690570	11.212303
H	8.374738	1.263201	11.187912
H	7.892604	2.033852	9.661673
C	10.153166	7.135089	11.422911
H	10.343126	6.683848	12.414071
H	9.604349	8.074254	11.573082
H	11.108325	7.374171	10.950645
C	7.994805	6.031180	11.054604
H	7.434785	5.418429	10.340558
H	7.496025	7.007720	11.140570
H	7.957720	5.551512	12.048057
C	13.553990	5.254953	6.293957
H	14.007088	5.376935	7.269685
C	13.697276	4.113893	5.453268
H	14.269490	3.219555	5.675280
C	12.970679	4.364444	4.244254
H	12.887747	3.692653	3.397148
C	12.383769	5.654456	4.353415
H	11.767881	6.135773	3.600885
C	12.749143	6.212847	5.610312
H	12.471913	7.197925	5.969104
C	10.304750	2.034809	8.214842
C	11.429252	1.297738	8.639728
C	11.452958	-0.072440	8.375680
H	12.324509	-0.650032	8.688558
C	10.398124	-0.722836	7.721701
C	9.298308	0.042391	7.327591
H	8.470499	-0.442477	6.806611
C	9.222909	1.421121	7.561753
C	12.583392	1.955849	9.350736
H	12.266944	2.396691	10.307071
H	13.016439	2.764576	8.745227
H	13.373284	1.227031	9.562514
C	10.443003	-2.208701	7.471417
H	9.864592	-2.483714	6.581588

H	10.017469	-2.759435	8.323729
H	11.472630	-2.561183	7.336768
C	8.015995	2.181884	7.069073
H	7.088305	1.667095	7.352893
H	8.019498	2.241916	5.972003
H	7.972270	3.206468	7.452973
C	11.049824	6.938272	8.258664
C	12.306498	7.392417	8.695007
C	12.674832	8.709435	8.385547
H	13.653934	9.067428	8.708776
C	11.832079	9.572120	7.681052
C	10.576985	9.087144	7.288205
H	9.895069	9.747137	6.748694
C	10.156216	7.785418	7.573312
C	13.257453	6.540518	9.502874
H	13.244595	6.834492	10.562984
H	14.290483	6.680163	9.160119
H	13.014025	5.474360	9.454872
C	12.244953	10.986519	7.360580
H	12.261651	11.158328	6.275411
H	13.240968	11.215747	7.755177
H	11.535544	11.708399	7.788168
C	8.767972	7.344933	7.188755
H	8.347420	8.008644	6.425229
H	8.101327	7.378999	8.063258
H	8.746327	6.320918	6.801605
C	10.831368	4.464784	7.788650
N	10.257750	3.445567	8.530041
N	10.636500	5.590677	8.575963
N	9.339386	3.092459	10.781646
N	9.359106	6.246184	10.572851
O	11.383353	1.498406	5.162525
O	8.931675	4.623809	4.681835

$[MnCp(CO)_2((IMes^{(NMe_2)_2})]^{2+}$ = $[5]^{2+}$ (triplet S=1 → -465.942 eV)

Note : both triplet (S=1 ; here) and BS (Ms=0 ; below) states have been geometry-optimized. They are quasi isoenergetic, meaning that both spin sources (Mn and NHC backbone) are isolated from one another (i.e. do not communicate with one another, magnetically speaking).

Mn	11.604509	4.387624	6.040636
C	10.039690	4.009047	5.204109
C	11.323487	6.097462	5.402653
C	9.771418	3.832382	9.860965
C	9.860357	5.254553	9.823010
C	9.652823	3.398921	12.282977
H	10.254180	4.309562	12.338073
H	10.222890	2.571998	12.731522
H	8.728211	3.525880	12.861909
C	8.696012	1.748056	10.721154
H	7.932037	1.661573	11.505930
H	9.406314	0.916112	10.834087
H	8.205198	1.683337	9.747981
C	9.938085	7.462652	10.997694
H	9.849790	7.636460	12.079108
H	9.399990	8.264415	10.471298
H	10.993908	7.492259	10.721332

C	8.064705	5.908906	11.376123
H	7.608882	4.979764	11.025736
H	7.392593	6.744975	11.132391
H	8.185613	5.879047	12.467300
C	13.495948	3.489520	6.619885
H	13.841790	3.366103	7.639252
C	12.758826	2.526784	5.859105
H	12.416098	1.556679	6.206237
C	12.568150	3.055474	4.551583
H	12.047702	2.564510	3.735227
C	13.174778	4.336824	4.496671
H	13.206083	4.988469	3.629392
C	13.753664	4.610235	5.779400
H	14.312142	5.502392	6.041964
C	10.526104	1.980154	8.322172
C	11.672976	1.358449	8.858555
C	11.913379	0.025984	8.509390
H	12.803978	-0.464137	8.906280
C	11.053867	-0.696367	7.668601
C	9.913115	-0.045905	7.186532
H	9.217956	-0.594164	6.548156
C	9.612381	1.284010	7.509839
C	12.621039	2.053856	9.805932
H	12.319214	1.893665	10.851968
H	12.670129	3.137673	9.645823
H	13.633659	1.645231	9.710387
C	11.328526	-2.138561	7.334005
H	10.876013	-2.425701	6.378217
H	10.903945	-2.796520	8.106917
H	12.404009	-2.345972	7.288941
C	8.297828	1.862525	7.046964
H	8.140785	1.684267	5.977089
H	8.209298	2.937970	7.228225
H	7.464113	1.365868	7.565607
C	10.744025	6.916199	8.143533
C	12.030002	7.475190	8.286082
C	12.215394	8.790318	7.848634
H	13.204524	9.239599	7.948525
C	11.172750	9.549801	7.301971
C	9.907412	8.953337	7.194927
H	9.084964	9.526366	6.764113
C	9.661701	7.641017	7.605006
C	13.168020	6.731272	8.939971
H	13.058523	6.705827	10.035506
H	14.122832	7.224871	8.729609
H	13.235314	5.690843	8.601284
C	11.403515	10.955034	6.817424
H	11.488149	10.971305	5.720472
H	12.325802	11.380406	7.227560
H	10.566616	11.612600	7.083010
C	8.287228	7.041918	7.440495
H	7.663661	7.680658	6.806485
H	7.766760	6.933930	8.403700
H	8.324685	6.046027	6.978330
C	10.761254	4.431371	7.877661
N	10.287951	3.379524	8.642818

N	10.506738	5.565198	8.622472
N	9.350235	3.052634	10.887266
N	9.356241	6.143676	10.714565
O	9.073459	3.806826	4.606108
O	11.202265	7.093905	4.840113

$[MnCp(CO)_2((IMes^{(NMe_2)_2})]^{2+} = [5]^{2+}$ (BS Ms=0 → -465.938 eV)

Mn	11.592669	4.377005	6.014325
C	9.967270	4.294387	5.219171
C	11.547074	6.134582	5.507733
C	9.773650	3.820815	9.837024
C	9.822939	5.251934	9.786210
C	9.597665	3.408188	12.260265
H	10.172010	4.335254	12.325928
H	10.180450	2.599438	12.725156
H	8.655791	3.509232	12.815133
C	8.742530	1.712058	10.689219
H	7.977646	1.599235	11.469374
H	9.485013	0.909952	10.810104
H	8.261163	1.625800	9.713055
C	9.820857	7.474105	10.937252
H	9.699527	7.664376	12.012419
H	9.264630	8.243446	10.382135
H	10.880897	7.537798	10.685160
C	8.003138	5.861961	11.329794
H	7.572500	4.919368	10.984214
H	7.313128	6.678861	11.072147
H	8.117665	5.847084	12.421511
C	13.436700	3.284679	6.450973
H	13.862872	3.144082	7.437679
C	12.558109	2.385792	5.778775
H	12.177995	1.445746	6.165683
C	12.279192	2.925418	4.491909
H	11.644260	2.473658	3.736226
C	12.992004	4.146949	4.353050
H	13.000784	4.784681	3.475348
C	13.709938	4.375560	5.571346
H	14.370497	5.213671	5.767743
C	10.600482	1.976379	8.344525
C	11.744954	1.384245	8.919370
C	12.004377	0.045322	8.615346
H	12.893541	-0.422840	9.040733
C	11.164627	-0.712199	7.784200
C	10.032512	-0.085548	7.253774
H	9.357833	-0.658411	6.615084
C	9.717289	1.253240	7.524477
C	12.671912	2.127894	9.850326
H	12.308328	2.091326	10.888386
H	12.784188	3.186038	9.582772
H	13.666483	1.668004	9.852544
C	11.457528	-2.162480	7.505803
H	10.954394	-2.510993	6.597253
H	11.104824	-2.789228	8.338400
H	12.533946	-2.343721	7.401286
C	8.430312	1.819891	6.977661
H	8.381423	1.709719	5.887296

H	8.288146	2.878501	7.214899
H	7.569609	1.265693	7.379324
C	10.711204	6.925607	8.135375
C	11.990701	7.486563	8.325562
C	12.180379	8.811220	7.926442
H	13.162862	9.263936	8.067305
C	11.150796	9.575110	7.357907
C	9.894542	8.974700	7.198507
H	9.082677	9.551708	6.753172
C	9.640645	7.654695	7.582556
C	13.118586	6.716408	8.965864
H	12.890855	6.436471	10.005946
H	14.033359	7.317573	8.984447
H	13.334783	5.785134	8.425483
C	11.394225	10.988326	6.904056
H	11.657733	11.003099	5.835618
H	12.221624	11.453237	7.451774
H	10.499697	11.610548	7.024526
C	8.265307	7.065416	7.391254
H	7.688537	7.662472	6.676999
H	7.693404	7.050713	8.331679
H	8.300874	6.035310	7.014488
C	10.783797	4.433003	7.862694
N	10.331956	3.374451	8.642132
N	10.471989	5.568893	8.595991
N	9.341374	3.043812	10.858691
N	9.292186	6.127760	10.672747
O	8.947272	4.263932	4.678297
O	11.590179	7.163767	4.988979
